# Novel Intrapolymerization Doped Manganese‐Eumelanin Coordination Nanocomposites with Ultrahigh Relaxivity and Their Application in Tumor Theranostics

**DOI:** 10.1002/advs.201800032

**Published:** 2018-03-25

**Authors:** Heng Liu, Chengchao Chu, Yu Liu, Xin Pang, Yayun Wu, Zijian Zhou, Pengfei Zhang, Weiguo Zhang, Gang Liu, Xiaoyuan Chen

**Affiliations:** ^1^ Department of Radiology the Third Affiliated Hospital Army Medical University Chongqing 400010 China; ^2^ State Key Laboratory of Molecular Vaccinology and Molecular Diagnostics & Center for Molecular Imaging and Translational Medicine School of Public Health Xiamen University Xiamen 361102 China; ^3^ Department of Ultrasound Southwest Hospital Army Medical University Chongqing 400000 China; ^4^ Laboratory of Molecular Imaging and Nanomedicine National Institute of Biomedical Imaging and Bioengineering National Institutes of Health Bethesda MD 20892 USA; ^5^ Chongqing Clinical Research Center for Imaging and Nuclear Medicine Chongqing 400010 China; ^6^ State Key Laboratory of Cellular Stress Biology Innovation Center for Cell Biology School of Life Sciences Xiamen University Xiamen 361102 China; ^7^ The MOE Key Laboratory of Spectrochemical Analysis & Instrumentation College of Chemistry and Chemical Engineering Xiamen University Xiamen 361005 China

**Keywords:** eumelanin, geometrical confinement, manganese, magnetic resonance imaging, theranostics

## Abstract

While magnetic resonance imaging contrast agents have potential in noninvasive image‐guided tumor treatment, further developments are needed to increase contrast, biodegradability, and safety. Here, novel engineered manganese‐eumelanin coordination nanocomposites (MnEMNPs) are developed via a facile one‐pot intrapolymerization doping (IPD) approach in aqueous solution, through simple chemical oxidation–polymerization of the 3,4‐dihydroxy‐DL‐phenylalanine precursor with potassium permanganate serving as the Mn source and an oxidant. The resulting MnEMNPs possess ultrahigh longitudinal relaxivity (*r*_1_ value up to 60.8 mM^−1^ s^−1^ at 1.5 T) attributed to the high manganese doping efficiency (>10%) and geometrically confined conformation. Due to their high manganese chelation stability, excellent biocompatibility, and strong near‐infrared absorption, high‐performance longitudinal‐transverse (*T*_1_ *‐T*_2_) dual‐modal magnetic resonance/photoacoustic imaging and photothermal tumor ablation are achieved. Furthermore, the hydrogen peroxide‐triggered decomposition behavior of MnEMNPs circumvents the poor biodegradation issue of many nanomaterials. This facile, convenient, economical, and efficient IPD strategy will open up new avenues for the development of high‐performance multifunctional theranostic nanoplatforms in bionanomedicine.

Among various medical imaging techniques, magnetic resonance imaging (MRI) is one of the most prevailing and prominent diagnostic modalities in clinic.^1^ To augment the diagnostic specificity, sensitivity and accuracy, many nanocomposites have been strategically exploited as MRI contrast agents (CAs).^2^, ^3^, ^4^, ^5^, ^6^, ^7^ Recently, efficient strategies to engineer longitudinal‐transverse (*T*_1_ *–T*_2_) dual‐modal CAs (DMCAs) have attracted considerable interest because they can provide complementary and synergistic diagnostic information over single modal imaging, conferring self‐confirmed false‐free merits with improved diagnostic accuracy.^8^, ^9^ Generally, the relaxivity of CAs could be improved by increasing the rotational correlation time (τ_R_), the number of water molecules coordinating to each metal ion (*q*), diffusion correlation time (τ_D_), and 1/water proton residence lifetime (τ_m_).^10^ Interestingly, geometrical confinement is an effective strategy for restricting the free rotation of CAs and diffusion of proximal water molecules. This increases τ_R_ and τ_D_, and further enhances *T*_1_ and *T*_2_ contrast.^10^, ^11^, ^12^

In addition to simple conjugation of *T*_1_ and *T*_2_ CAs, concentrated *T*_1_ components can be incorporated into nonmagnetic porous matrices (e.g., polymer,^13^ albumin nanoparticle,^14^ mesoporous silica,^12^ metal organic framework,^15^ and hydrogel^16^) to obtain *T*_1_ *–T*_2_ DMCAs. Here, the coordination and chemical exchange of the surrounding protons are restricted by the so‐called geometrical confinement effect due to the unique architecture,^12^ which results in enhanced *T*_1_ contrast. Restricted water diffusion due to the concentration effect could disturb the motional averaging effect of *T*_2_ relaxation and offer improved *T*_2_ contrast.^16^ Developing nanoagents that are solely composed of naturally occurring components in organisms via convenient procedures are of high benefit for advanced biomedical applications due to their ability to undergo decomposition into nontoxic metabolites and thus demonstrate minimal adverse effects during their retention in vivo.^17^ Melanins, generally categorized into eumelanin, pheomelanin, and neuromelanin, are ubiquitous pigment biomacromolecules in various parts of living organisms.^18^ The excellent biocompatibility/biodegradability and fascinating biological properties of melanin‐like nanoparticles (MelNPs) make them highly intriguing for various biomedical applications,^19^ such as photothermal agents,^20^ photoacoustic CAs,^21^, ^22^ drug delivery,^23^ and antioxidative therapy against ischemic stroke.^24^ Recently, various paramagnetic metal ions (e.g., iron,^13^, ^25^, ^26^, ^27^, ^28^ gadolinium,^29^ and manganese^23^, ^30^, ^31^) have been anchored onto MelNPs as alternative MRI CAs without the need for extrinsic chelators because of melanins' excellent metal ion chelating capacity. However, they are usually first synthesized via chemical or enzymatic oxidation‐polymerization of appropriate precursor molecules (e.g., dopamine, tyrosine, and 3,4‐dihydroxy‐L‐phenylalanine) under strong basic conditions (e.g., sodium hydroxide, ammonium hydroxide) and organic solvents.^13^, ^20^, ^30^ The transition metal ions are then chelated onto the as‐synthesized MelNPs (designated as postpolymerization doping strategy, PPD). This not only complicates the anchoring procedures and purification processes, but also suffers from low metal loading efficiency (weight/weight, wt/wt, less than 1%) and a risk of chelated ion detachment from the surface.^25^ Furthermore, few of them have adequate water dispersibility. Rational engineering of water‐dispersible MelNPs with satisfactory contrast via convenient procedures is highly attractive but remains challenging.^13^

In this work, we introduce a novel one‐pot intrapolymerization doping (IPD) strategy to fabricate water‐dispersible manganese‐eumelanin coordination nanocomposites (MnEMNPs). The as‐prepared MnEMNPs possess ultrahigh relaxivity (*r*_1_ value up to 60.8 mM^−1^ s^−1^ at 1.5 T, ≈8.9 times higher than that of clinical gadolinium‐based CAs) attributed to the high Mn doping efficiency (>10%) and geometrically confined conformation. The PEGylated MnEMNPs (denoted as PMnEMNPs; PEG is polyethylene glycol) demonstrated satisfactory results for *T*_1_ *–T*_2_ dual‐modal MRI/photoacoustic imaging (PAI) and photothermal tumor ablation (**Scheme** **1**). Furthermore, the good biocompatibility and hydrogen peroxide–triggered decomposition behavior of MnEMNPs circumvent the poor biodegradation issue of many nanomaterials, holding great potential as a promising candidate in clinical translation. This facile, convenient, economical, and efficient IPD strategy would open up new avenues for the development of high‐performance multifunctional theranostic nanoplatforms in bionanomedicine.

**Scheme 1:**
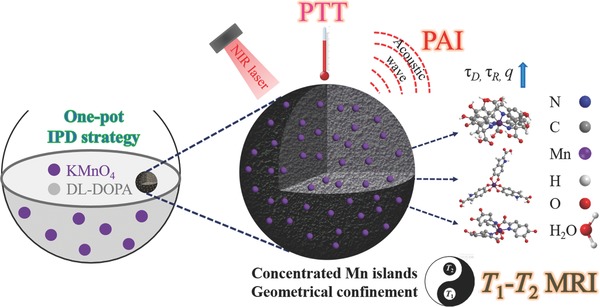
Synthesis procedure and theranostic applications of MnEMNPs. The DL‐DOPA precursor self‐polymerizes into MnEMNPs via a one‐pot IPD strategy. The KMnO_4_ serves as the Mn source and an oxidant concurrently. Mn ions with more than one coordinating water molecule (*q*) are abundantly incorporated into MnEMNPs. The conformation of MnEMNPs creates a geometrically confined space resulting in prolonged τ_R_ and τ_D_. These contributors enhance the *T*_1_ contrast of MnEMNPs. The as‐obtained MnEMNPs were explored for *T*_1_ *–T*_2_ dual‐modal MRI/PAI and tumor PTT.

The MnEMNPs were prepared through simple chemical oxidation‐polymerization of the 3,4‐dihydroxy‐DL‐phenylalanine (DL‐DOPA) precursor with potassium permanganate (KMnO_4_) serving as the Mn source and an oxidant concurrently. It likely involves the oxidation of catechol to benzoquinone, intramolecular cyclization through Michael reaction to generate indole, polymerization to eumelanin, and Mn chelation.^19^ During polymerization, low valence states of Mn ions reduced from KMnO_4_ are continuously incorporated into MnEMNPs accompanied by nanostructure formation. This is attributed to chemical interaction with abundant anchoring sites, resulting in several coordination species (Figure S1, Supporting Information).^29^ This IPD strategy simplifies the fabrication procedures without the need for any additional chelation processes or extrinsic chelators compared with conventional PPD approaches. This is crucial for large‐scale controllable synthesis of products with reproducible yields and properties among multiple batches.

Optimization of experimental conditions and detailed properties characterization results are shown in Table S1 and Figures S2–S8 (Supporting Information). The DL‐DOPA/KMnO_4_ feeding molar ratio of 1:0.3 was an optimal reaction parameter to obtain the largest amount of water‐dispersible eumelanin nanoparticles with well‐defined spherical morphology. This was used for typical synthesis and subsequent experiments. In a typical synthesis, approximately 50 mg products could be obtained, determined by weighing after lyophilization. The Mn loading efficiency reached 10.2% wt/wt (Table S1, Supporting Information), which is much higher than previous studies.^13^, ^29^, ^32^, ^33^ It suggests more efficient synthesis and higher yields than conventional PPD approaches. The as‐obtained MnEMNPs remained stable for six months without any detectable agglomeration, demonstrating good water‐dispersibility and dispersion stability. The MnEMNPs demonstrated well‐organized shape uniformity with tightly stacked structure on transmission electron microscopy (TEM) images due to the interaction of oligomeric units (**Figure** **1**a).^34^ There was a characteristic broad single‐line electron spin resonance (ESR) spectrum in accordance with that of previously reported melanins (Figure 1b). It originates from the π‐electron free radical sites in eumelanin.^35^ The Raman spectrum exhibited two characteristic band signals located near 1387 cm^−1^ and 1590 cm^−1^ (Figure 1c) due to the vibration of sp^2^‐bonded carbon atoms that is almost identical to that of natural eumelanins.^36^ The results from TEM, ESR, and Raman spectrum are almost identical to natural and synthetic MelNPs suggesting the successful synthesis of artificial eumelanin nanospheres. The scanning electron microscope (SEM) energy dispersive X‐ray element mapping (Figure 1d) and line scanning (Figure S9, Supporting Information) data confirmed the presence of Mn component within MnEMNPs. X‐ray photoelectron spectrometer (XPS) was performed to analyze the valence states of the elements (Figure S10, Supporting Information). The characteristic peaks centered at 641.5 and 653.65 eV were assigned to Mn 2p_3/2_ and Mn 2p_1/2_ (Figure 1e), respectively, indicating the presence of a large portion of Mn (II) and a tiny fraction of Mn (III) species. Notably, the Mn content in MnEMNPs exhibited almost no loss after one week (Figure 1f), indicating its high stability and negligible Mn detachment. This is crucial to minimize the risk of released‐Mn‐ion‐associated biotoxicity.

**Figure 1:**
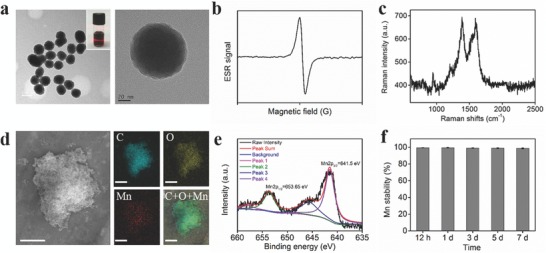
Characterization of MnEMNPs. a) TEM images. Scale bars: 100 nm (left) and 20 nm (right). The inset shows digital photograph of MnEMNPs aqueous solution. b) ESR spectra. c) Raman spectrum. d) SEM images and corresponding elemental mapping images (C, O, and Mn). Scale bar, 5 µm. e) Mn2p_1/2_ and Mn2p_3/2_ XPS spectra. f) Mn stability inside MnEMNPs, determined by ICP‐MS.

The concentrated Mn components inside MnEMNPs and their superior stability motivated us to explore their feasibility as MRI CAs due to their inherent paramagnetic nature.^37^ Field‐dependent magnetic‐hysteresis (M‐H) curve of MnEMNPs exhibited a linearly increased magnetization upon applied magnetic field (MF) with negligible coercivity and remanence at room temperature (**Figure** **2**a), suggesting their paramagnetic behavior. The low saturated magnetization (0.66 emu g^−1^) indicated negligible local MF generation and minimal *T*_2_ decay effect. To understand the coordination environment and structure‐activity relationship, the ^1^H nuclear magnetic relaxation dispersion (NMRD) profiles of MnEMNPs were determined from 4 to 62 MHz (Figure 2b,c). Different species populations with different coordination environments and intraparticle magnetic interactions (Figure S1, Supporting Information) lead to a relatively complicated shape.^30^, ^38^ The calculated relaxivity increased in a proton Larmor frequency‐dependent manner with a plateau at 42.5 and 55 MHz for longitudinal (*r*_1_) and transverse (*r*_2_) relaxivity, respectively. The shape and amplitude suggested that more than one water molecule could interact with the metal centers.^32^

**Figure 2:**
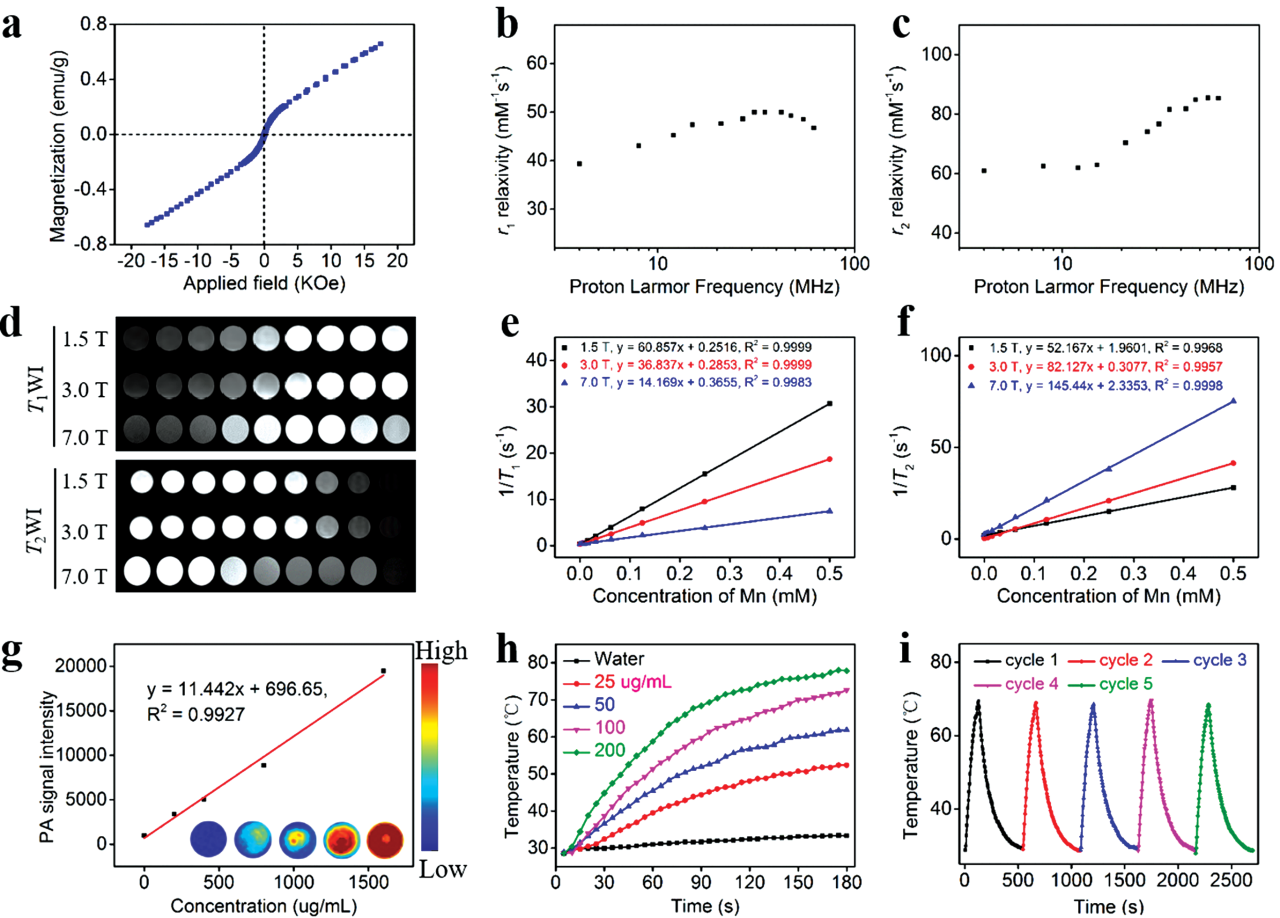
Magnetic/photoacoustic/photothermal performance of MnEMNPs. a) M‐H curve at 300 K. b,c) ^1^H NMRD profiles. d) *T*_1_WI and *T*_2_WI at various MFs. The linear relationship for the e) *r*_1_ and f) *r*_2_ relaxivities of MnEMNPs as a function of Mn concentration at various MFs. g) PA signal intensity under 800 nm as a function of the concentration of MnEMNPs. The insets show corresponding PA images of MnEMNPs solution. h) Temperature elevation of MnEMNP solutions with various concentrations during 2 W cm^−2^ laser irradiation. i) Photothermal heating curves of 100 µg mL^−1^ MnEMNPs under 2 W cm^−2^ laser irradiation over five laser on/off cycles.

To further evaluate the relaxation profiles of MnEMNPs, their *r*_1_ and *r*_2_ relaxivities were measured at a wide range of MFs. Both *T*_1_WI and *T*_2_WI demonstrated prominent concentration‐dependent contrast enhancement (Figure 2d). For a given metal ion concentration, the MnEMNPs provided better *T*_1_ contrast than commercial gadopentetate dimeglumine (Gd‐DTPA) and Mn ion standards at the same MF (Figure S11, Supporting Information). At 1.5 T, the *r*_1_ relaxivity was up to 60.8 mM^−1^ s^−1^, approximately 8.9‐fold higher than Gd‐DTPA and 8‐fold higher than Mn ion standards, respectively. The relaxivity of MnEMNPs far surpasses that of most previously reported metal ion incorporated MelNPs (Table S2, Supporting Information) and manganese‐based MRI CAs (Table S3, Supporting Information). The significantly improved relaxivity of MnEMNPs may come from the following contributors: (i) the high Mn loading provides concentrated paramagnetic islands that may interact with nearby paramagnetic centers during one relaxation process, thus facilitates proton coordination and chemical exchange; (ii) several intermediate species of MnEMNPs can embrace Mn ions to form a geometrically confined conformation due to the swollen effect. This would restrict the rotation of Mn ions and the diffusion of surrounding water molecules resulting in prolonged τ_R_ and τ_D_. This improves the *r*_1_ relaxivity (Scheme 1). The *r*_1_ values were 36.8 and 14.2 mM^−1^ s^−1^ at 3.0 and 7.0 T, respectively (Figure 2e). This was attributed to the interference via *T*_2_ decay effect. An elevated MF would induce the proton Larmor frequency in excess of the molecular vibration frequency resulting in confining spin‐lattice relaxation and decreased *r*_1_ relaxivity. The *r*_2_ values were 52.2, 82.1, and 145.4 mM^−1^ s^−1^ at 1.5, 3.0, and 7.0 T, respectively (Figure 2f). This could be explained by the magnetic susceptibility effect resulting from the concentrated Mn ions entrapped in MnEMNPs. The relaxivity variations at different MFs (from 1.5 to 9.4 T) resulted in an increase in the *r*_2_/*r*_1_ ratio from 0.86 to 10.79 (Table S4, Supporting Information). Taken together, the results suggested the potential of MnEMNPs as novel high‐performance *T*_1_ *–T*_2_ DMCAs.

Notably, the reaction solution exhibited broad optical absorption and increased with time during the reaction process (Figure S12, Supporting Information), presumably attributed to the oxidation and subsequent polymerization of DL‐DOPA.^39^ The MnEMNPs demonstrated concentration‐dependent optical absorption (Figure S13a, Supporting Information) and their absorbance at 808 nm linearly correlated with mass concentrations (Figure S13b, Supporting Information), suggesting their potential for PAI and photothermal therapy (PTT) applications.^21^, ^22^, ^40^, ^41^, ^42^ As expected, the MnEMNPs solution exhibited concentration‐dependent PA signals (Figure 2g) and the solution temperature gradually increased with concentration, laser power density, and irradiation time (Figure 2h, Figure S14 in the Supporting Information). No significant decrease in temperature variation was observed over five laser irradiation cycles (Figure 2i). After longstanding laser irradiation, the MnEMNPs demonstrated much better photothermal stability than Au nanorods (Figure S15, Supporting Information), confirmed by TEM and optical absorption, suggesting great potential for PTT applications.

To improve dispersion stability of MnEMNPs for biological applications, they were PEGylated by linkage via a Michael addition or Schiff base reaction.^43^ There was no obvious NIR absorption change after PEGylation (**Figure** **3**a). The average hydrodynamic sizes of MnEMNPs and PMnEMNPs were 144 and 162.5 nm (Figure 3b), respectively. The slightly larger hydrodynamic size of MnEMNPs than on TEM images was likely due to the surrounding water molecules and the swollen effect.^20^ The surface zeta potentials of MnEMNPs and PMnEMNPs were −33.2 and −39.3 mV (Figure 3c), respectively, which were sufficient to ensure excellent colloidal stability. The Fourier transform infrared (FT‐IR) peak at 1082 cm^−1^ was assigned to the C—O—C stretching of mPEG‐SH on PMnEMNPs (Figure 3d). The dynamic light scattering and FT‐IR results indicated successful PEGylation of MnEMNPs. The absorbance of MnEMNPs decreased to a different extent after incubation in various physiological media for 24 h (Figure S16, Supporting Information), while no apparent change for PMnEMNPs (Figure 3e), suggesting much improved colloidal stability after PEGylation (Figure S17, Supporting Information). Hemolytic activity was observed at 200 µg mL^−1^ MnEMNPs, which was not seen by PMnEMNPs (Figure 3f), indicating more favorable hemocompatibility of PMnEMNPs. The poor biodegradability of many nanomaterials is a critical obstacle in their clinical translation.^44^ The tumor cells often produce more elevated H_2_O_2_ levels than that in healthy cells,^45^, ^46^ and thus H_2_O_2_ was used to imitate the intrinsic tumor characteristics. The PMnEMNPs solution demonstrated a lighter color and decreased absorbance in the presence of H_2_O_2_ (Figure S18, Supporting Information), indicating their specific and substantial disintegration sensitive to H_2_O_2_.^20^ The nanostructure gradually collapsed and dissociated into smaller residues with increasing H_2_O_2_ concentrations (Figure 3g), and almost completely destroyed after 24 h in 10 × 10^−3^ m H_2_O_2_. These features give PMnEMNPs tumor‐responsive degradability and good biocompatibility, making them suitable and safe for bioapplications.

**Figure 3:**
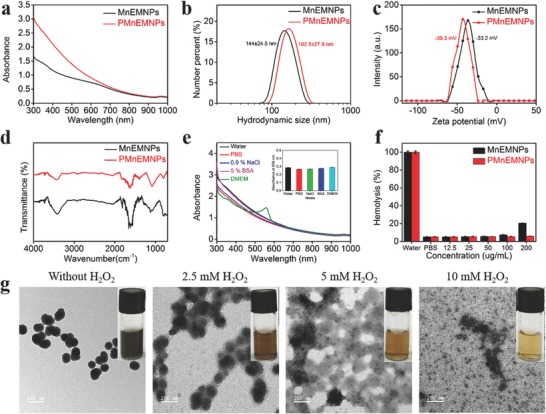
PEGylation and H_2_O_2_‐responsive decomposition behavior of NPs. a) UV–vis absorption spectra, b) hydrodynamic size, c) zeta potential, and d) FT‐IR spectra. e) UV–vis absorption spectra of PMnEMNPs dispersed in various media for 24 h. The inset shows corresponding absorbance at 808 nm. f) Hemolysis analysis. g) TEM images showing the structural evolution of PMnEMNPs in the presence of H_2_O_2_ for 24 h. Scale bar, 200 nm. The insets show corresponding digital photographs.

Subsequently, the cellular uptake, cell cytotoxicity, and in vitro photothermal cytotoxicity against tumor cells were assessed. Cellular labeling assay showed plentiful yellow particles in U87MG cells accompanied by a clean background (**Figure** **4**a), which were found to be incorporated into endosome‐like structures in the cytoplasm (Figure 4b). The Mn content in cells increased with incubation concentration (Figure 4c), quantified by inductively coupled plasma mass spectrometry (ICP‐MS). These results suggested efficient binding and internalization of PMnEMNPs by U87MG cells. Cellular PA images demonstrated incubation concentration‐dependent signal intensities (Figure 4d). The collected cells also exhibited significant positive and negative contrast enhancement on *T*_1_WI and *T*_2_WI, respectively. Both the *T*_1_ and *T*_2_ relaxation time of cells were significantly shortened in an incubation concentration‐dependent manner (Figure 4e,f). The cell MRI/PAI results suggested that the internalized PMnEMNPs could improve the visualization of tumor cells. An ideal theranostic agent should be low‐toxic for bioapplications. The thiazolyl blue tetrazolium bromide results showed negligible cytotoxicity of PMnEMNPs on U87MG cells at the tested concentrations (Figure 4g) indicating excellent biocompatibility. However, the cell viability significantly decreased with increased PMnEMNPs incubation concentrations following laser irradiation (Figure 4h), verifying their effective photothermal cytotoxicity. The Calcein acetoxymethyl ester/propidium iodide (AM/PI) staining results also confirmed the high performance of PMnEMNPs (Figure 4i) as photothermal agents for tumor treatment.

**Figure 4:**
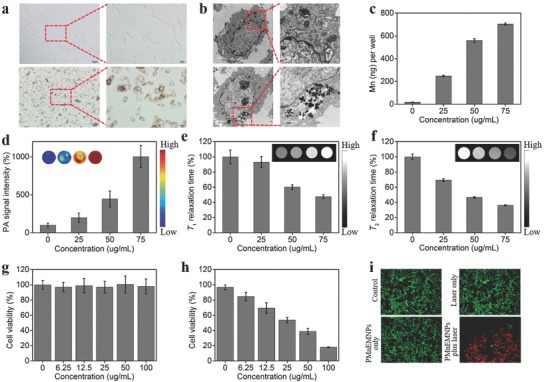
In vitro cellular uptake and theranostic results of PMnEMNPs. a) Representative optical microscope and b) TEM images of U87MG cells incubated without (upper row) or with (lower row) PMnEMNPs. Scale bars: 100 and 50 µm for panel (a), 2 and 1 µm for panel (b), respectively. c) Quantitative cellular uptake of PMnEMNPs by ICP‐MS analysis. d) PA signal intensities of U87MG cells after incubation with different concentrations of PMnEMNPs. The insets show corresponding PA images. Normalized e) *T*_1_ and f) *T*_2_ relaxation times of U87MG cells after incubation with different concentrations of PMnEMNPs. The insets in panels (e) and (f) show corresponding *T*_1_WI and *T*_2_WI, respectively. The viability of U87MG cells after g) incubation with different concentrations of PMnEMNPs and h) exposed to PMnEMNPs plus 2 W cm^−2^ laser irradiation for 5 min. i) Calcein‐AM/PI co‐stained fluorescence images of U87MG cells that received different treatments.

To explore the biocompatibility of PMnEMNPs in vivo, serum biochemical analysis (Figure S19, Supporting Information) and hematoxylin‐eosin staining (Figure S20, Supporting Information) were performed, indicating their suitability and safety for in vivo applications via intravenous administration.^20^ All animal experiments were performed in accordance with the protocol approved by the Animal Care and Use Committee of Xiamen University, China. Encouraged by the brilliant MRI results of PMnEMNPs in vitro, their bioaccumulation and biodegradation profiles were preliminarily investigated by monitoring time‐resolved MR signal intensity changes in the liver (Figure S21, Supporting Information) and kidneys (Figure S22, Supporting Information). The specific and rapid decomposition and excretion behaviors of PMnEMNPs would minimize the poor biodegradation issue of many nanomaterials. Note that manganese is an essential microelement for physiological processes, and melanins are widely distributed in the living subjects and can be physically metabolized. This ensures the biosafety of PMnEMNPs.

Considering the excellent biocompatibility and high contrast capabilities of PMnEMNPs, in vivo MR/PA imaging were then performed for tumor accumulation assessment. The PMnEMNPs provided positive and negative contrast enhancement on *T*_1_WI and *T*_2_WI (**Figure** **5**a), respectively. Both *T*_1_ (Figure 5b) and *T*_2_ relaxation time (Figure 5c) in the tumor region were significantly shortened at 10 min postadministration due to the first‐pass effect. Subsequently, they both reached a minimum at 2 h postinjection at about 70% and 85%, respectively. Such a change was attributed to the selective tumor accumulation of PMnEMNPs, and then the *T*_1_ and *T*_2_ relaxation times gradually recovered to about 86% and 91% at 24 h postinjection, respectively. Representative in vivo PA images were presented in Figure 5d. The PA signal intensity in the tumor region reached a maximum at 2 h postinjection (Figure 5e) consistent with the MRI results. The PA signal then gradually recovered and was close to that of baseline at 24 h postinjection suggesting further metabolic processes of PMnEMNPs. The results demonstrated the efficient tumor accumulation of PMnEMNPs for tumor visualization, attributed to the well‐known enhanced permeability and retention (EPR) effect. Next, tissue TEM was performed for direct observation of the decomposition behavior of PMnEMNPs inside the tumor with elevated H_2_O_2_ levels. Initially, PMnEMNPs were readily internalized by tumor cells and maintained an intact nanostructure morphology (Figure S23, Supporting Information). Subsequently, their decomposition was clearly shown with prolonged time, revealed by a collapsed morphology without defined spherical structures and fusion of degraded products. The intracellular degradation results agreed well with aforementioned biodegradation assay in vitro. This suggests that the intracellular elevated H_2_O_2_ levels could trigger PMnEMNPs disintegration to minimize the poor biodegradation issue of many nanomaterials.

**Figure 5:**
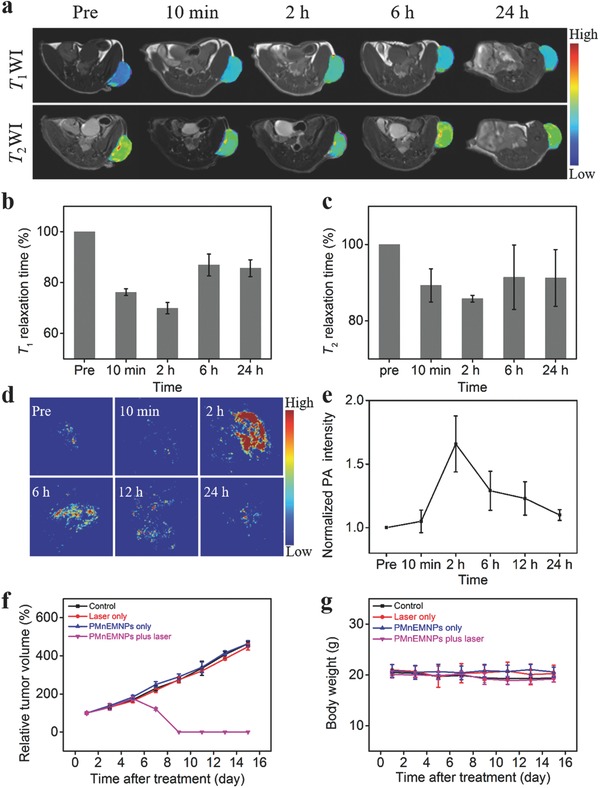
In vivo theranostic evaluation of PMnEMNPs. a) In vivo *T*_1_WI and *T*_2_WI, b) normalized *T*_1_, and c) *T*_2_ relaxation times of U87MG tumor‐bearing mouse prior to and at different time points postinjection of PMnEMNPs. d) PA images and e) corresponding normalized PA signal intensity from the tumor at different time points. f) Tumor growth curves and g) body weight of mice from different treatment groups.

Encouraged by the effective accumulation of PMnEMNPs in tumor tissues and their outstanding photothermal cytotoxicity in vitro, their antitumor efficacy on tumor‐bearing mice was investigated. The temperature elevation at tumor site positively correlated with laser irradiation time in mice treated with PMnEMNPs (Figure S24, Supporting Information). At 24 h posttreatment, the destroyed structure inside the tumor tissues in PMnEMNPs group was observed by extensive tissue edema and cell necrosis (Figure S25, Supporting Information). There was no obvious destruction in the control groups. Tumor growth profiles were monitored for 15 days to directly evaluate the therapeutic effects. Effective inhibition and even eradication of tumor growth were achieved in the PMnEMNPs group (Figure 5f, Figures S26 and S27 in the Supporting Information), while negligible tumor suppression was observed in the other groups, suggesting the potent photothermal efficacy of PMnEMNPs for tumor treatment. Notably, the body weight of mice remained constant during the evaluation period (Figure 5g), suggesting no obvious side effect due to treatment.

In summary, novel engineered multifunctional MnEMNPs are developed as a theranostic nanoplatform for *T*_1_ *–T*_2_ MRI/PAI and photothermal tumor ablation. The specific novelties of this work include: (i) simple fabrication of water‐dispersible MnEMNPs via a facile one‐pot IPD strategy in aqueous phase through simple chemical oxidation‐polymerization of DL‐DOPA with KMnO_4_ serving as the Mn source and an oxidant concurrently; (ii) ultrahigh Mn loading efficacy (>10%) is achieved without the need for any additional chelation process or extrinsic chelator; (iii) remarkably improved *r*_1_ (*r*_1_ value up to 60.8 mM^−1^ s^−1^ at 1.5 T) and *r*_2_ relaxivities attributed to the high manganese doping efficiency and geometrically confined conformation; and (iv) specific decomposition behavior that is sensitive to high H_2_O_2_, evading the poor biodegradation issue for many nanomaterials. The biocompatible and decomposable PMnEMNPs with superior imaging and therapy capabilities are a promising candidate for efficient tumor theranostics. This facile, convenient, economical, and efficient IPD strategy will open up new avenues for the development of high‐performance multifunctional theranostic nanoplatforms in bionanomedicine.

## Conflict of Interest

The authors declare no conflict of interest.

## Supporting information

Supplementary
