# Supplementary material for: Novel Intrapolymerization Doped Manganese‐Eumelanin Coordination Nanocomposites with Ultrahigh Relaxivity and Their Application in Tumor Theranostics
Source: Adv Sci (Weinh). 2018 Mar 25;5(7):1800032. doi: 10.1002/advs.201800032 (PMC6051206; doi:10.1002/advs.201800032)
Supplement: Supplementary file 1 — Supplementary [file ADVS-5-1800032-s001.pdf]

Copyright WILEY-VCH Verlag GmbH & Co. KGaA, 69469 Weinheim, Germany, 2018.

ADVANCED SCIENCE Open Access

Supporting Information

for *Adv. Sci.,* DOI: 10.1002/advs.201800032

Novel Intrapolymerization Doped Manganese-Eumelanin Coordination Nanocomposites with Ultrahigh Relaxivity and Their Application in Tumor Theranostics

Heng Liu, Chengchao Chu, Yu Liu, Xin Pang, Yayun Wu, Zijian Zhou, Pengfei Zhang, Weiguo Zhang, Gang Liu,* and Xiaoyuan Chen*

Copyright WILEY-VCH Verlag GmbH & Co. KGaA, 69469 Weinheim, Germany, 2016.

Supporting Information

Novel Intra-polymerization Doped Manganese-eumelanin Coordination Nanocomposites with Ultrahigh Relaxivity and Their Application in Tumor Theranostics

Heng Liu, Chengchao Chu, Yu Liu, Xin Pang, Yayun Wu, Zijian Zhou, Pengfei Zhang, Weiguo Zhang, Gang Liu,* and Xiaoyuan Chen*

H. Liu, Prof. W. Zhang
Department of Radiology, the Third Affiliated Hospital, Army Medical University, Chongqing, 400010, China. E-mail: wgzhang01@163.com
H. Liu, C. Chu, Y. Liu, X. Pang, Y. Wu, Dr. Z. Zhou, P. Zhang, Prof. G. Liu
State Key Laboratory of Molecular Vaccinology and Molecular Diagnostics & Center for Molecular Imaging and Translational Medicine, School of Public Health, Xiamen University, Xiamen, 361102, China. E-mail: gangliu.cmitm@xmu.edu.cn
Y. Liu
Department of Ultrasound, Southwest Hospital, Army Medical University, Chongqing, 400000, China.
Dr. Z. Zhou, Dr. X. Chen
Laboratory of Molecular Imaging and Nanomedicine, National Institute of Biomedical Imaging and Bioengineering, National Institutes of Health, Bethesda, Maryland 20892, USA.
Prof. W. Zhang
Chongqing Clinical Research Center for Imaging and Nuclear Medicine, Chongqing, 400010, China.
Prof. G. Liu
State Key Laboratory of Cellular Stress Biology, Innovation Center for Cell Biology, School of Life Sciences, Xiamen University, Xiamen, 361102, China.
Prof. G. Liu

The MOE Key Laboratory of Spectrochemical Analysis & Instrumentation, College of Chemistry and Chemical Engineering, Xiamen University, Xiamen, 361005, China.

EXPERIMENTAL SECTION

Reagents. 3,4-dihydroxy-DL-phenylalanine (DL-DOPA), potassium permanganate ($KMnO_4$), Mn ions standards, PI (propidium iodide) and Calcein AM (calcein acetoxymethyl ester) were obtained from Aladdin Reagent (Los Angeles, Southern California, USA). Thiol-terminated methoxy-poly (ethylene glycol) (mPEG-SH, MW=5000 Da) was purchased from Seebio (Shanghai, China). Ammonia solution, dimethyl sulphoxide (DMSO) and thiazolyl blue tetrazolium bromide (MTT) were obtained from Sigma Aldrich (St Louis, MO, USA). Gadopentetate dimeglumine (Gd-DTPA, Magnevist) was purchased from CONSUN (Guangzhou, China). All other chemicals, unless otherwise noted, were analytical grade and used as received without further purification. Deionized water used in the experiment was purified by a Millipore system.

Synthesis of MnEMNPs and PEGylation. Synthesis of manganese-eumelanin coordination nanocomposites (MnEMNPs) was carried out by chemical oxidation-polymerization of DL-DOPA with $KMnO_4$ in aqueous solution. In a typical synthesis, DL-DOPA (60 mL, 10 mM) suspension was heated to 50 °C. Then, $KMnO_4$ solution (1.8 mL, 100 mM) (DL-DOPA/$KMnO_4$ feeding molar ratio was 1:0.3) received ultrasonic dispersion previously was rapidly added under vigorous stirring. Upon $KMnO_4$ added, the colorless solution became dark purple and then gradually turned to deep black as the reaction progressed. The reaction was allowed to

proceed under vigorous stirring for 6 h. Then, the products were retrieved by several sequential centrifugation/redispersion processes in deionized water (17500 rpm, 15 min), to remove excess precursors and reactants. Finally, the as-obtained MnEMNPs were redispersed in deionized water. The yields of MnEMNPs were determined by weighing after lyophilization and the Mn content was quantified by inductively coupled plasma mass spectrometry (ICP-MS) following digestion by aqua regia overnight.

To optimize the reaction conditions, the effect of reaction time and DL-DOPA/$KMnO_4$ feeding molar ratio on the nanostructure formation were systematically investigated. Detailed reaction parameters for the synthesis of MnEMNPs are summarized in Table S1. The same synthetic procedure were performed for MnEMNPs with 1:0.15 (Condition I), 1:0.3 (Condition II), and 1:0.6 (Condition III) DL-DOPA/$KMnO_4$ feeding molar ratios, respectively, adopting constant amounts of DL-DOPA and different amounts of $KMnO_4$.

PEGylated MnEMNPs (hereafter denoted as PMnEMNPs) were prepared by mixing MnEMNPs with mPEG-SH at a feeding mass ratio of 1:5 in alkaline buffer solution (pH = 9.8-10.3) under vigorous stirring overnight at room temperature.[1] The product was purified by centrifugation and washed several times with deionized water to remove residual mPEG-SH. The as-obtained PMnEMNPs were redispersed in water for future use.

Physicochemical characterization. UV-vis spectra of reaction solution was monitored using an UV-vis spectrometer (Cary60, Agilent Technologies, USA). The morphology of MnEMNPs was characterized by transmission electron microscopy (TEM, Hitachi H-7500, Tokyo, Japan). Electron spin resonance (ESR) spectra was determined by a JEOL JES-TE200 apparatus. Raman spectrum was recorded on a Raman spectrometer with 530 nm wavelength incident laser light. Energy-dispersive X-ray (EDS) elemental mapping, line-scanning and SEM images were captured on a field emission scanning electron microscope (SEM, Hitachi SU-70, Tokyo, Japan). The valence states of manganese component were analyzed by X-ray photoelectron spectrometer (XPS). To determine the stability of incorporated Mn ions in MnEMNPs, MnEMNPs solutions (100 μg Mn mL^{-1}, three replicates) were added in Eppendorf tubes and let stand at room temperature. At selected time points, supernatants (200 μL) were harvested by centrifugation (15000 rpm, 15 min) for ICP-MS analysis. The hydrodynamic sizes and surface zeta potentials were measured by dynamic light scattering (DLS, Zetasizer Nano ZS90, Malvern Instruments, UK). The chemical functional groups were characterized by fourier transform infrared spectroscopy (FTIR, Bruker, Karlsruhe, Germany).

For photoacoustic contrast enhancement measurement, MnEMNPs (200 μL) with various concentrations were filled into eppendorf tubes to produce signals upon 800 nm laser excitation, using a photoacoustic tomography imaging system (Nexus128, Endra Life Sciences, MI, USA). To assess the photothermal efficacy of MnEMNPs, 200 μL MnEMNPs aqueous solution with different concentrations (0-200 μg mL^{-1})

were irradiated by 808 nm laser (2 W cm^{-2}, 3 min). Deionized water was set as control. The solution temperature was recorded using an infrared thermal camera. MnEMNPs (200 μg mL^{-1}) aqueous solution were irradiated by 808 nm laser at various power densities (0.5-2.5 W cm^{-2}) for 3 min. The photothermal stability of MnEMNPs was assessed at 100 μg mL^{-1} via cyclic-irradiation assay (2 W cm^{-2}, laser irradiation for 2 min and then shut off for 7 min). To compare the photothermal stability of MnEMNPs and Au nanorods. MnEMNPs and Au nanorods (100 μg mL^{-1}) were irradiated by 808 nm laser (2 W cm^{-2}, 30 min), the changes of optical absorption and morphology were measured by UV-vis spectrometer and TEM, respectively.

MRI relaxivity measurement. Magnetic property measurement was performed using a vibrating sample magnetometer (VSM) (Quantum, USA) at 300 K. Proton $1/T_1$ and $1/T_2$ NMRD profiles at various magnetic fields in the range of 0.09-1.45 T (corresponding to 4-62 MHz proton Larmor frequencies) were measured on a fast field-cycling relaxometer (Stelar, Italy) at room temperature. To measure the longitudinal (r_1) and transverse (r_2) relaxivity, MnEMNPs were dispersed in ultrapure water with various metal molar concentrations (0-0.5 mM), using Gd-DTPA and Mn ion standards as control. Phantom images were acquired using a 7.0 T small animal MR scanner (Bio-Spec, Bruker, Karlsruhe, Germany) at room temperature with the following acquisition parameters: (1) T_1 RARE sequence: TR/TE: 1500/8 ms; echo spacing: 8 ms; averages: 4; slice thickness: 1 mm, matrix: 256 × 256; FOV: 2.5 × 2.5 cm; (2) T_1-map sequence: TR ranges from 447 ms to 5,500 ms; TE: 8.5 ms; echo spacing: 8.5 ms; echo images: 10; slice thickness: 1 mm; matrix: 256 × 256; FOV: 2.5

× 2.5 cm; (3) Turbo RARE-T_2 sequence: TR/TE: 2,500/35 ms; echo spacing: 11.5 ms; averages: 4; slice thickness: 1 mm, matrix: 256 × 256; FOV: 2.5 × 2.5 cm; (4) T_2-map MSME sequence: TR: 4,500 ms; TE ranges from 9.5 ms to 237.5 ms; echo spacing: 9.5 ms; echo images: 25; slice thickness: 1 mm; matrix: 256 × 256; FOV: 2.5 × 2.5 cm. The r_1 and r_2 relaxivity were obtained using 1/relaxation time (s^{-1}) plotted against metal concentrations (mM) and calculated by a linear fit, respectively. The MRI relaxivity under various magnetic fields was also measured using a 9.4 T small animal MR scanner (Bio-Spec, Bruker, Karlsruhe, Germany), a 3.0 T clinical scanner (Siemens, Magnetom Verio, Munich, Germany), a 1.5 T HT-MICNMR-60 benchtop relaxometer (Huantong Corporation, Shanghai, China), and a 0.5 T NMI20-Analyst NMR Analyzing & Imaging system (Niumag Corporation, Shanghai, China), respectively.

Colloidal stability, hemocompatibility and degradability assay. To investigate the colloidal dispersion stability of NPs in various biological media, MnEMNPs and PMnEMNPs (100 μg mL^{-1}) were dispersed in water, PBS, 0.9% NaCl, 5% BSA and DMEM, respectively. The mixing solutions were kept stand at room temperature. At selected time intervals, the digital photos were taken, and then 100 μL of the upper solution was collected carefully for UV-vis spectra measurement.

For hemolysis analysis, fresh blood samples from healthy Blab/c mice were washed two times with cold PBS. Then, the red blood cells (RBCs) were carefully isolated by centrifugation (3000 rpm, 10 min) and diluted in PBS (v/v, 0.25%) and incubated with

various concentrations of MnEMNPs or PMnEMNPs at 37 ℃ for 10 h. The final concentrations of NPs were 12.5, 25, 50, 100, and 200 μg mL^{-1}, respectively. After centrifugation (15000 rpm, 5 min), supernatants (200 μL) were collected and their absorbance at 541 nm were determined. Diluted RBCs suspension incubated with deionized water and PBS were set as the positive and negative control, respectively. Hemolysis activity was calculated using the following formula: Hemolysis% = $(OD_{sample}-OD_{water})/(OD_{PBS}-OD_{water})$, Where OD is the absorbance of samples at 541 nm. Hemolysis% > 5% indicated hemolytic activity.

To evaluate the degradation profiles *in vitro*, PMnEMNPs (100 μg mL^{-1}) were suspended into PBS (pH=7.4) without or with H_2O_2 (2.5, 5.0 and 10.0 mM), respectively. The solution was kept stand at room temperature. At selected time intervals, digital photos of the solution were taken, and then solution (100 μL) was collected for detecting the accumulated degradation content using an UV-vis spectrometer. TEM images were obtained for intuitive observation of time-dependent structural evolution during degradation process.

Cell experiments. Human glioblastoma cells (U87MG) were obtained from the Type Culture Collection of the Chinese Academy of Sciences (Shanghai, China). For cytotoxicity assay, U87MG cells were seeded in a 96-well plate with 5x10^{3} cells per well and incubated overnight at 37 ℃. PMnEMNPs with various concentrations were incubated with cells for 24 h. A standard MTT assay was performed to determine cell viability (%), calculated as the absorbance percentage of the control cells.

For cellular uptake assay, U87MG cells were incubated with PMnEMNPs (50 μg mL^{-1}) for 6 h. After that, cells were washed with PBS for several times and observed by an optical microscope. For bio-TEM, cells were collected by trypsinization and fixed in 2.5% cold glutaraldehyde, and then 1% aqueous OsO_4, dehydrated, embedded, and sectioned. The ultrathin sections were stained with lead citrate and uranyl acetate for TEM observation. For ICP-MS quantification, following incubation with various concentrations of PMnEMNPs for 6 h, the collected cells were digested with aqua regia at 37 °C overnight. Then, the digested solutions were diluted with 2% HNO_3 and the Mn content was determined by ICP-MS, using Mn ion standards for establishing standard curves.

For *in vitro* cell imaging, $2x10^6$ U87MG cells were seeded in cell culture dishes and allowed to 85% confluence. Then, various concentrations of PMnEMNPs were incubated with cells for 6 h. After washed three times with PBS, the cells were digested by trypsin, collected and suspended in 1% low melting agarose (200 μL). *In vitro* cell MR and cell PA images were acquired using a 7.0 T small animal MRI scanner and NEXUS 128 PAI system, respectively.

For *in vitro* photothermal cytotoxicity, U87MG cells were seeded in a 12-well plate with $5x10^4$ cells per well and incubated overnight at 37 °C. PMnEMNPs (50 μg mL^{-1}) were incubated with cells for 6 h. Then, cells were washed with PBS for three times and replaced with fresh medium, following by 808 nm laser irradiation (2 W cm^{-2}, 5 min). After incubation for another 4 h, cells were co-stained with Calcein AM and PI

for 30 min. The cells were visualized using a fluorescence microscope, which live and dead cells exhibited green and red fluorescence, respectively. To quantify the photothermal cytotoxicity of PMnEMNPs, $5x10^3$ U87MG cells were seeded in a 96-well plate. PMnEMNPs with various concentrations were incubated with cells for 6 h. Then, cells were washed with PBS and exposed to 808 nm laser (2 W cm^{-2}, 5 min), and then incubated with fresh media for another 24 h. Cell viability was determined by standard MTT assay.

Bioaccumulation of PMnEMNPs. T_1- and T_2- weighted MR images containing coronal and axial planes were acquired sequentially prior to and at different time points post intravenous injection of PMnEMNPs (73 μmol Mn kg^{-1} BW), using a 7.0 T MR scanner with the imaging parameters as mentioned earlier. The signal intensity was determined by analyzing the region of interest (ROI) to quantify the contrast enhancement.

PMnEMNPs (20 mg kg^{-1} BW) was intravenously injected into U87MG tumor-bearing mice. At selected time points, the mice were sacrificed, and the major organs and tumor tissues were collected and weighed before chemical digestion in trace metal grade 80% nitric acid (HNO_3) and 20% hydrogen peroxide (H_2O_2) for 24 h at 37 ℃. Subsequently, the samples were diluted with 2% HNO_3 and filtered through a 0.22 μm membrane. The distribution of PMnEMNPs was evaluated by quantifying the Mn content by ICP-MS analysis, recorded as nanogram Mn per milligram tissue (ng mg^{-1}).

Serum biochemical analysis. Blood was extracted from healthy mice that intravenously administered with PMnEMNPs (20 mg kg^{-1} BW) at different time points. Mice that received saline treatment were set as control. In order to separate serum, fresh blood samples were collected *via* the ocular vein (0.8-1.0 mL for each mouse), then centrifuged twice (3000 rpm, 10 min). The important hepatic and renal indicators were measured using a Biochemical Autoanalyzer (Type 7170, Hitachi, Japan).

Establishment of tumor model. Balb/c nude mice (female, 4-5 weeks, 20 ± 2 g) were supplied by Center for Experimental Animals, Xiamen University, China. All animal experiments were performed in accordance with the protocol approved by the Animal Care and Use Committee of Xiamen University, China. Tumor model was established by subcutaneous injection of 10^6 U87MG cells suspended in 100 μL PBS into the left back of mice. They were used for further experiments when the tumor volume reached 100 mm^3, which about three weeks after tumor inoculation.

***In vivo* theranostic evaluation of PMnEMNPs.** For *in vivo* MR/PA imaging, U87MG tumor bearing mice were anesthetized by 10 wt% chloral hydrate, and then PMnEMNPs (200 μL) aqueous solution was intravenously injected into mice (73 μmol Mn kg^{-1} BW). T_1WI and T_2WI were acquired before and at different time points post injection of PMnEMNPs using a 7.0 T MR scanner. T_1 and T_2 relaxation times were measured with the same sized ROI on the same slice of T_1-map and T_2-map images, respectively. To eliminate the influence of tumor variations among mice,

normalized T_1 and T_2 relaxation times were calculated as a function of post-injection time and by setting that of prior to injection as 100%. PA images were acquired using NEXUS 128 PAI system.

For *in vivo* tumor photothermal therapy, when the volume of tumors reached about 100 mm^3, mice were randomly divided into four groups with four mice each, minimizing tumor size and body weight differences. There were PBS treated (group I), laser only (group II), PMnEMNPs only (group III), and PMnEMNPs plus laser (group IV), respectively. Group III and IV were intravenously injected with PMnEMNPs (20 mg kg^{-1} BW), group II and IV were irradiated by 808 nm laser (2 W cm^{-2}, 15 min) at 2 h post-injection. Thermal images at tumor site were obtained using an infrared thermal camera. For observation of *in vivo* therapeutic effect, the body weight and tumor volume of mice were recorded every other day. Tumor size was measured using a caliper, and tumor volume was calculated according to the formula: tumor volume = $width^2$ * length/2.

For histological analysis, hematoxylin and eosin (H&E) staining, Ki-67 and TUNEL immunohistochemical staining were performed at 24 h after treatment. The mice were sacrificed on the 15th day post treatment, and their main organs and tumor were harvested and fixed in 4% formalin for 24 h. The tissues were then paraffin embedded and sectioned into a thickness of 5 μm. The sections were stained and then visualized using an optical microscope.

Statistical analysis. All data were presented as mean ± standard deviation. Statistical significance was determined by a two-tailed Student's t test assuming equal variance using SPSS 19.0. P value < 0.05 was considered statistically significant.

Supplementary Results

The PEGylation of MnEMNPs was confirmed by FT-IR spectra (Figure 3c). Characteristic peaks from 3300 cm^{-1} to 3500 cm^{-1} were attributed to N-H and O-H stretching vibrations. The peak at 1617 cm^{-1} was assigned to aromatic C=C bonds. *Versus* MnEMNPs, the peaks on PMnEMNPs at 2922 cm^{-1} and 2851 cm^{-1} were assigned to alkyl C-H stretching of mPEG-SH. The new peak at 1082 cm^{-1} was attributed to the C-O-C stretching of mPEG-SH on PMnEMNPs indicating successful PEGylation.

AM/PI staining was performed to visualize the photothermal killing effect. As shown in Figure 4i, cells treated with either laser irradiation only or PMnEMNPs only showed similar green fluorescence compared to the control suggesting negligible cell death. In contrast, cells treated with PMnEMNPs plus laser irradiation exhibited very little green fluorescence but vivid red fluorescence accompanied by decreased cell counts and floating cells.

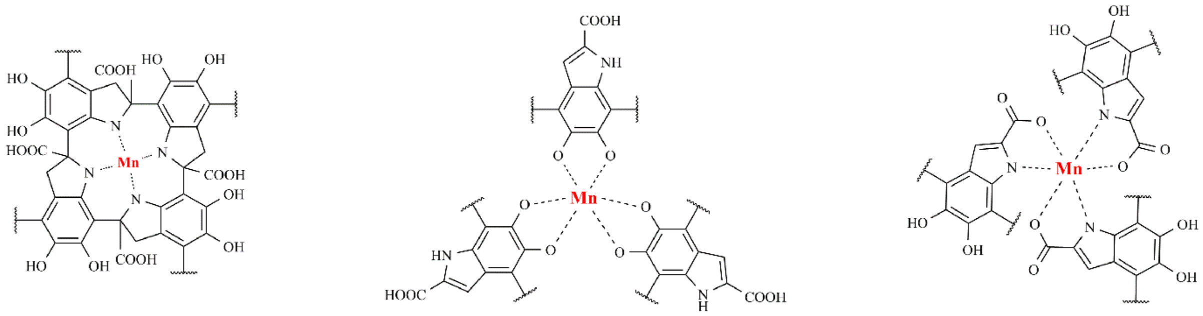

Supplementary Figure 1. Schematic illustration of three possible coordination species.

Time-dependent UV-vis absorption spectra of the reaction solution (diluted 10 times) with different reaction conditions were monitored (Figure S2). During the reaction process, the absorbance of the reaction solution gradually increased with time, and the absorbance growth was slower and reached a maximum at 50 ^{o}C for 6 h, probably due to the embedded DL-DOPA and oligomers inside the nanoparticles.

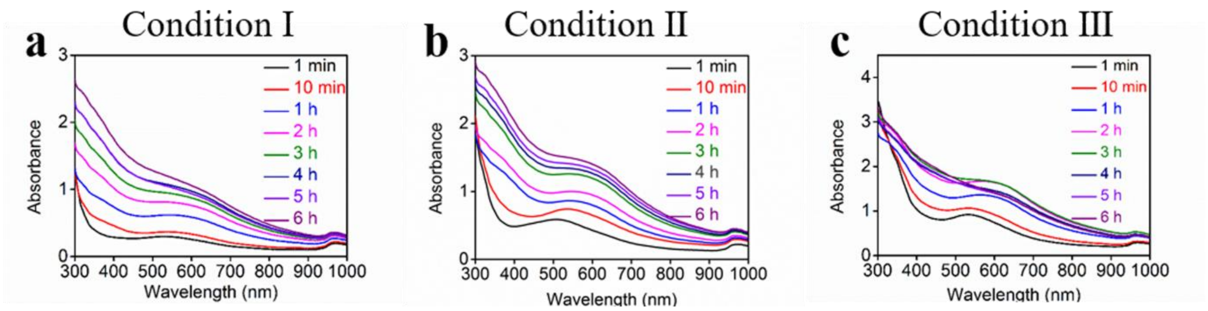

Supplementary Figure 2. Time-dependent UV-vis absorption spectra of the reaction solution (diluted for 10 times) with different reaction conditions.

TEM images of products with different reaction conditions demonstrated that the products were cross-linked and tightly stacked together for Condition III (Figure S3). The products in the other two groups exhibited well-defined spherical morphology. The size and morphology of MnEMNPs were similar to naturally occurring melanins, which is 40-150 nm in diameter and spherical in shape.

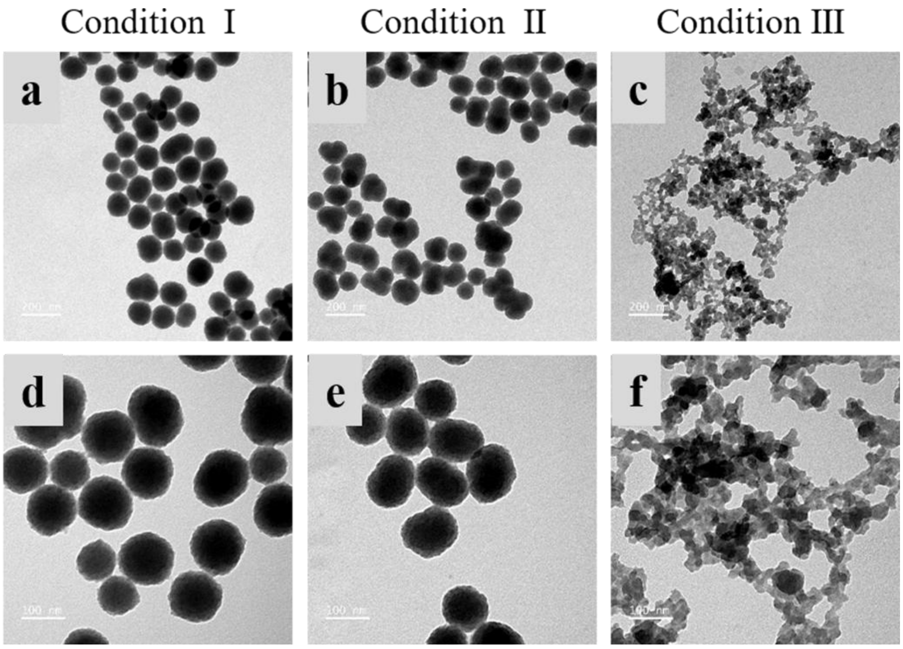

Supplementary Figure 3. TEM images of products with different reaction conditions. Scale bar, 200 nm (a-c) and 100 nm (d-f).

Figure S4 shows DLS data of MnEMNPs with different reaction conditions. The average hydrodynamic sizes were about 145.4, 148.3, and 218.2 nm for Condition I-III, respectively. The smaller hydrodynamic diameters of NPs for Conditions I and II may result from their excellent monodispersity in water, which is consistent with TEM results. The zeta potentials were about -37.9, -37.4, and -28.5 mV for Conditions I-III, respectively.

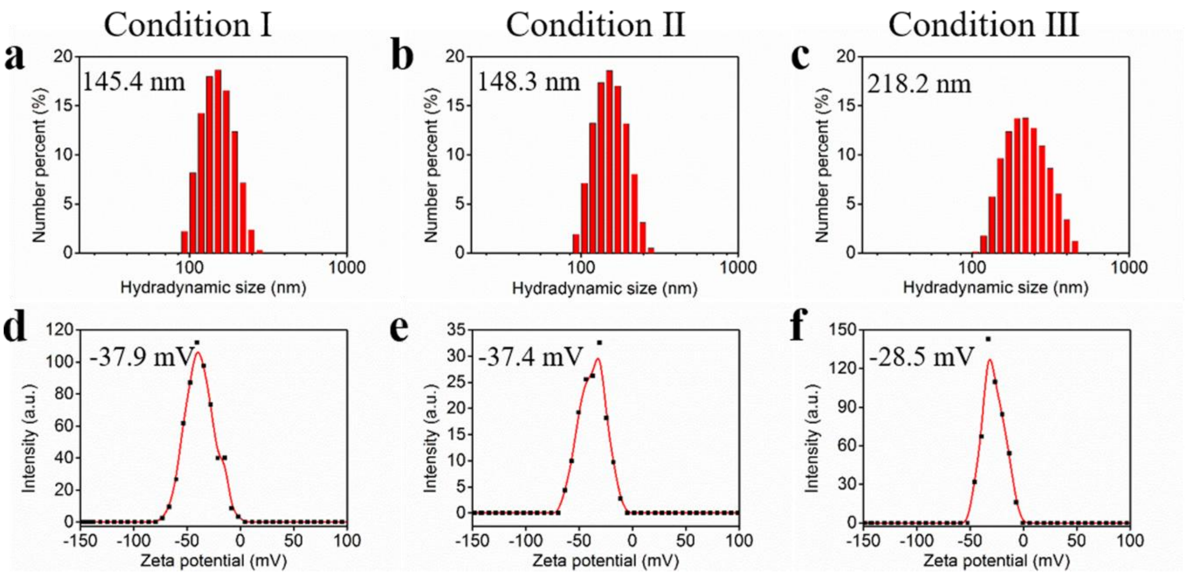

Supplementary Figure 4. DLS data of products with different reaction conditions. (a-c) Average hydrodynamic size. (d-f) Zeta potential.

Figure S5 showed UV-vis absorption spectra and the linear relationship for the absorbance at 808 nm as a function of the concentration of MnEMNPs with different reaction conditions. All UV-vis absorption spectra gradually increased in a concentration-dependent manner across the testing concentration range. There was a significant positive correlation between the absorbance at 808 nm and the concentration of MnEMNPs for each reaction group. The slope was much gentler for Condition III than the other two reaction groups. This is attributed to the cross-link and poor formation of the products. The strong NIR absorption of MnEMNPs indicated their potential for PAI and PTT applications.

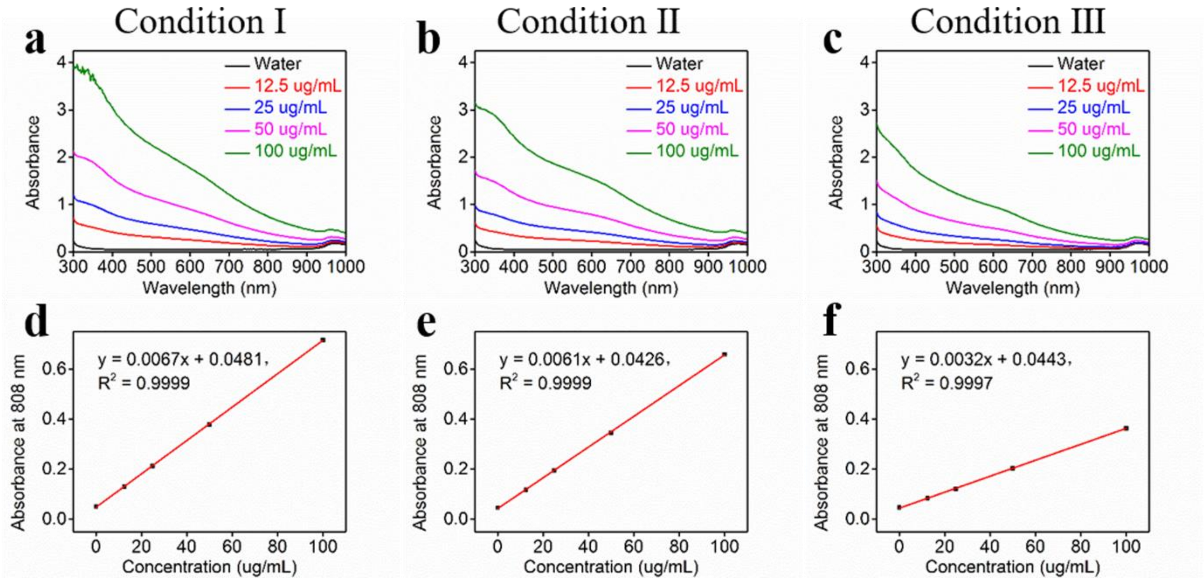

Supplementary Figure 5. UV-vis absorption spectra (a-c) and the linear relationship for the absorbance at 808 nm as a function of the concentration of products with different reaction conditions (d-f).

Figure S6 indicates the linearity of PA signal intensity under 800 nm as a function of the concentration of MnEMNPs with different reaction conditions. The PA signal intensity of the products gradually increased in a concentration-dependent manner in the testing concentration range. There was a significant positive correlation between the PA signal intensity under 800 nm and the concentration of MnEMNPs for each reaction group. The slope was much steeper for Condition II than the other reaction groups.

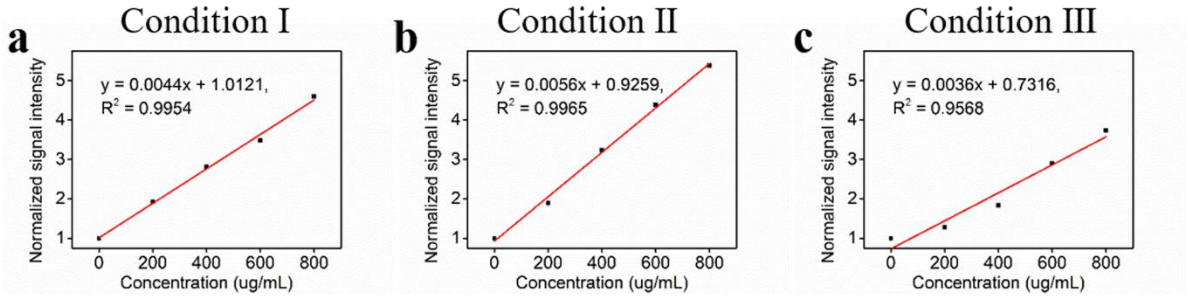

Supplementary Figure 6. The linearity of the PA signal intensity under 800 nm as a function of the concentration of products with different reaction conditions.

Figure S7 showed photothermal performance of MnEMNPs with different reaction conditions. When continuously exposed to a 808 nm laser at a power density of 2 W cm^{-2}, the temperature of the MnEMNPs aqueous solutions dramatically increased in both a concentration- and irradiation time-dependent manners.

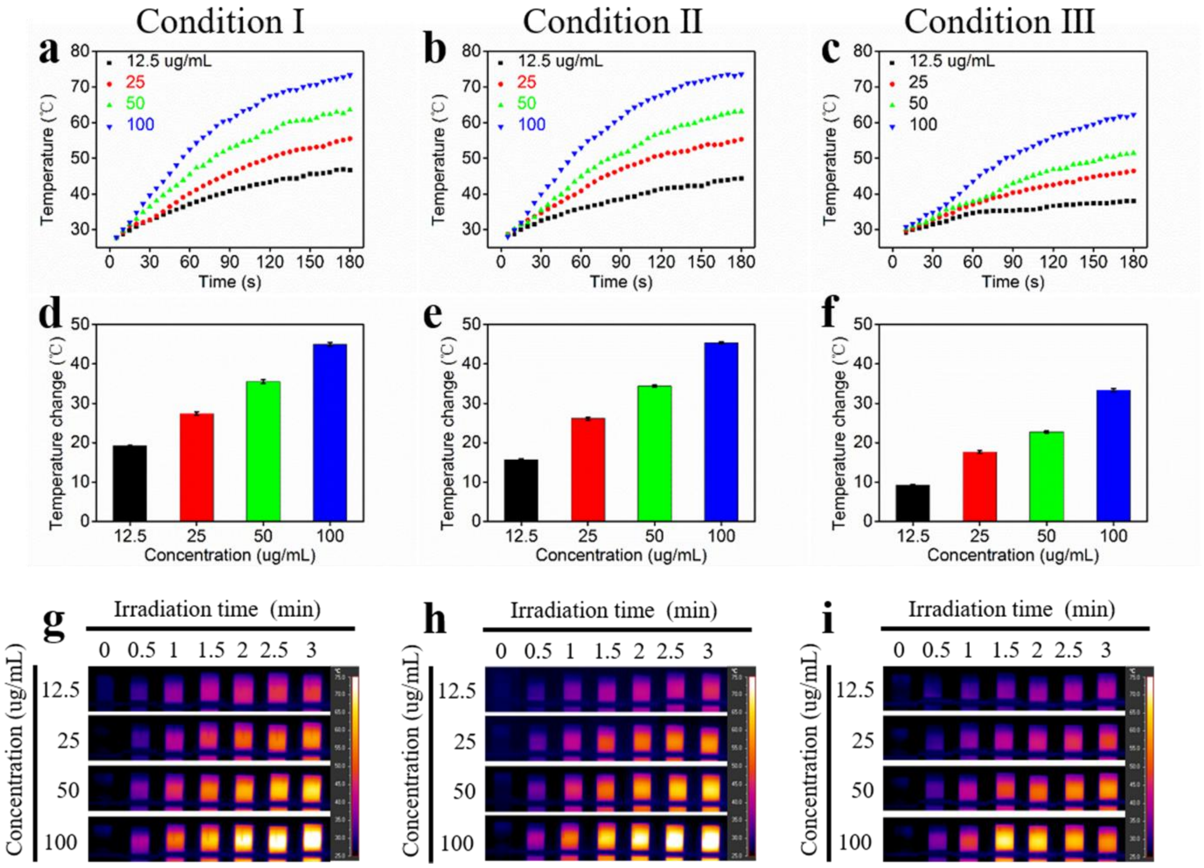

Supplementary Figure 7. Photothermal performance of products with different reaction conditions. Temperature elevation (a-c), final temperature change (d-f), and corresponding thermal images (g-i) of products with various concentrations as a function of irradiation time with exposure to the 808 nm laser at 2 W cm^{-2}. The error bars are the mean values ± s.d.; n = 3.

Figure S8 showed MR phantom images and corresponding linear relationship for the r_1 and r_2 relaxivity as a function of Mn concentration at 7.0 T with different reaction conditions. The r_1 relaxivities were determined to be 13.55, 14.17, and 10.28

$mM^{-1}s^{-1}$, respectively (a-c). The r_2 relaxivities were 125.7, 145.44, and 117.79 $mM^{-1}s^{-1}$, respectively (d-f).

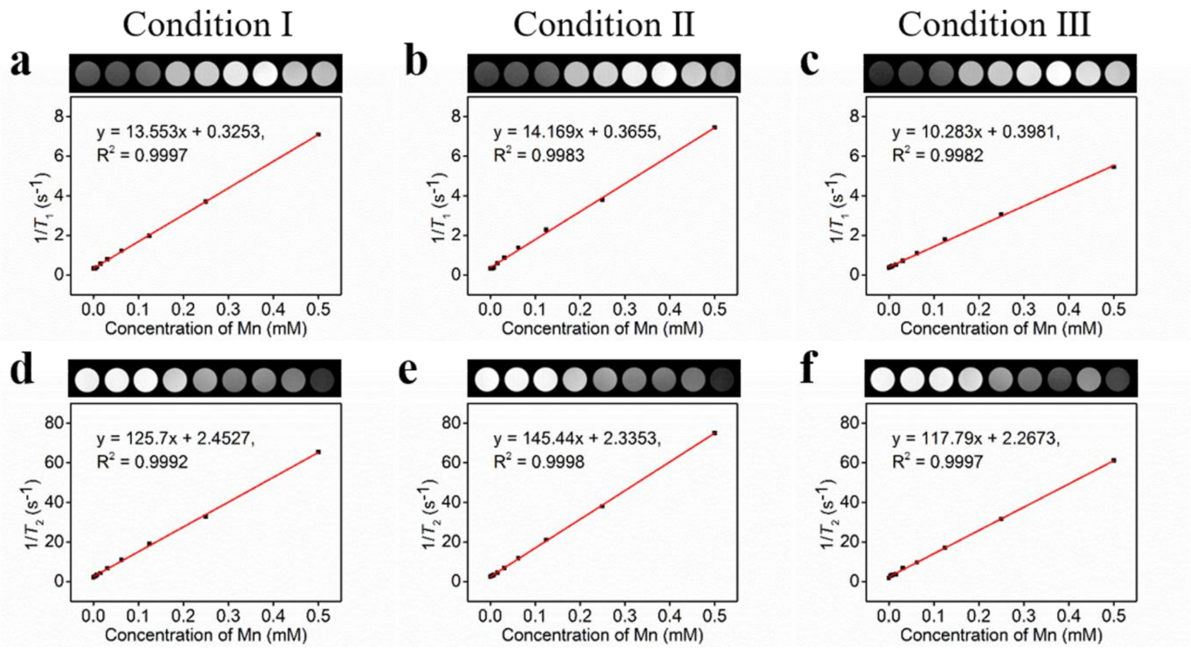

Supplementary Figure 8. MR phantom images and corresponding linear relationship for the r_1 and r_2 relaxivity of products as a function of the Mn concentration at 7.0 T with different reaction conditions. (a-c) T_1-weighted images (upper) and r_1 relaxivity (lower). (d-f) T_2-wieighted images (upper) and r_2 relaxivity (lower).

The optimal reaction parameter would be at DL-DOPA/$KMnO_4$ feeding molar ratio of 1:0.3 after considering Mn loading efficiency, yields, physicochemical characteristics, and photoacoustic/photothermal/magnetic performance of MnEMNPs. Because of their suitable size and excellent properties, this type of MnEMNPs was selected for typical synthesis and subsequent experiments.

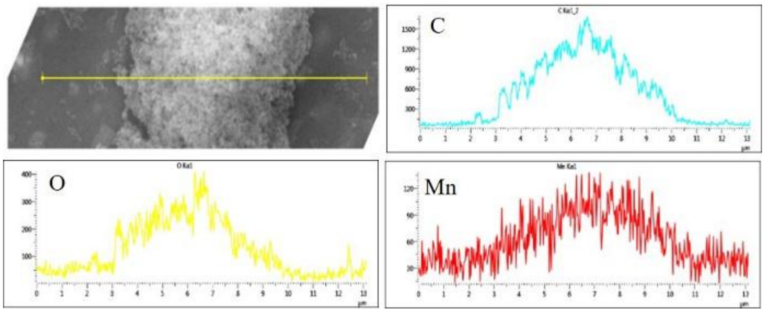

Supplementary Figure 9. SEM-EDS Linear scan results of MnEMNPs.

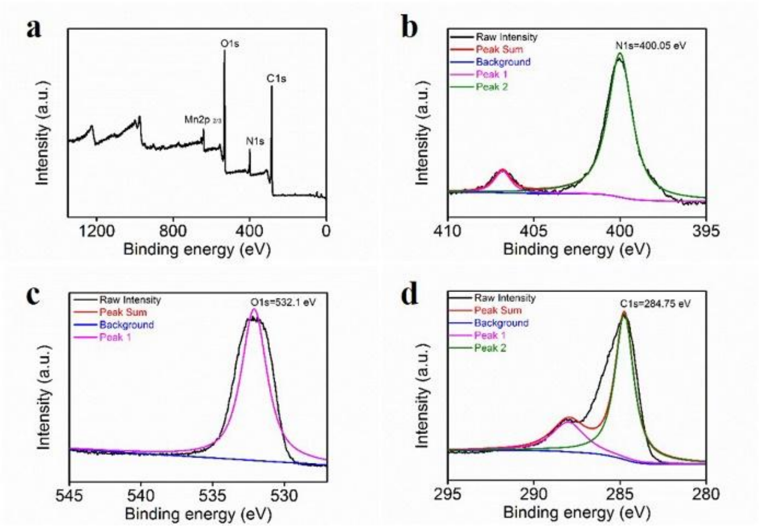

Supplementary Figure 10. XPS spectra of MnEMNPs. (a) XPS survey spectra. (b) N1s, (c) O1s, and (d) C1s spectra.

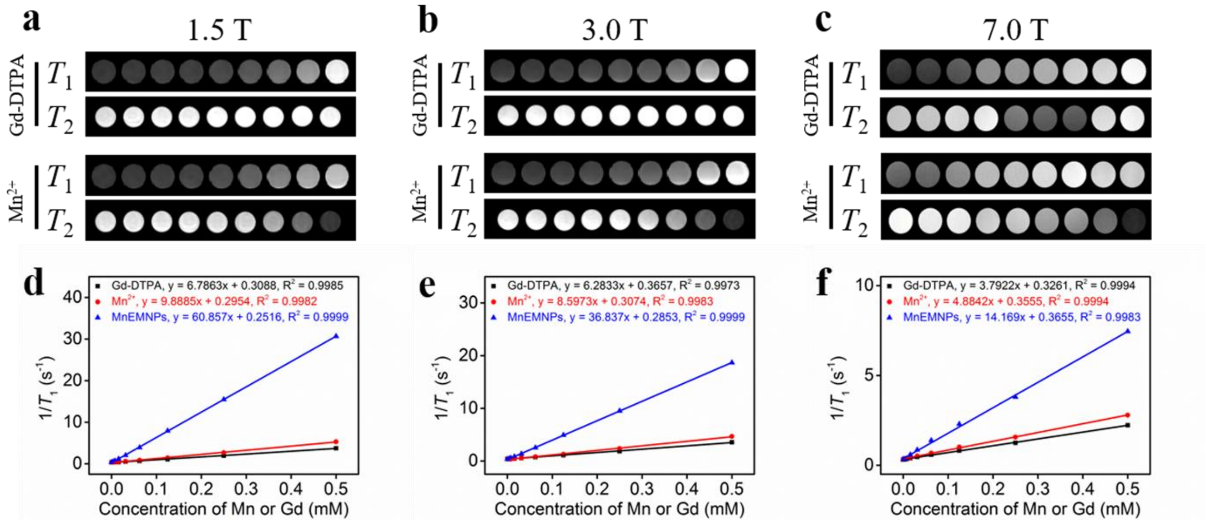

Supplementary Figure 11. Relaxivity measurement. T_1WI and T_2WI of commercialized Gd-DTPA and Mn^{2+} standards and corresponding linear relationship for the r_1 relaxivity as a function of metal ion concentration at various MFs.

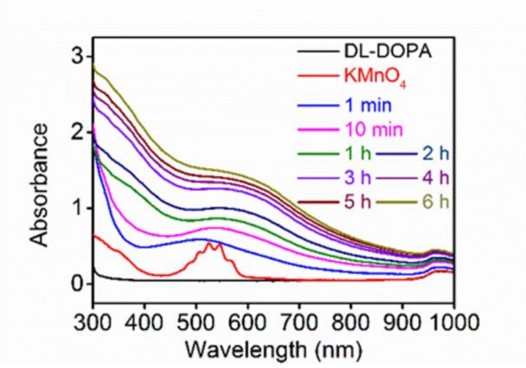

Supplementary Figure 12. Time-dependent UV-vis absorption spectra of the reaction solution.

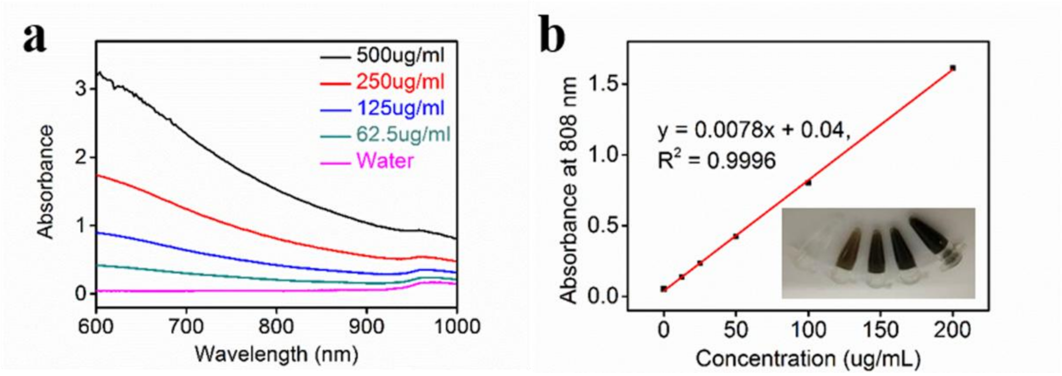

Supplementary Figure 13. (a) UV-vis absorption spectra of MnEMNPs with different mass concentrations. (b) The linearity for absorbance at 808 nm as a function of the concentration of MnEMNPs. The insets in Figure b show corresponding photographs of MnEMNPs solution.

Calculation of photothermal conversion efficacy (PTCE, defined as η) of MnEMNPs at 808 nm. During the cooling period (Figure S14d), the driving force temperature (θ) was determined according to Equation 1: $\theta = (\mathrm{T} - \mathrm{T}surr)/(\mathrm{T}max - \mathrm{T}surr)$, where T, T_{surr}, and T_{max} is the solution temperature, ambient temperature of the surroundings, and the equilibrium temperature, respectively. Here, τ_s, the time constant for heat transfer from the sample system and was determined to be $\tau_s = 142.37\ s$, by applying linear time data from the cooling period *vs* negative natural logarithm of θ (-Ln θ) (Figure S14e). Here, *hS* is a dimensionless driving force temperature and was determined according to Equation 2:$hS = m_s C_p/\tau s$, where m_s (0.2 g) and C_p (4.2 J g^{-1}) are the mass and heat capacity of pure water, respectively. Substituting τ_s value into Equation 2, *hS* was

determined to be 7.11 mJ. The η of MnEMNPs at 808 nm was calculated according to Equation 3 as previously described:[2] $\eta = (hS \Delta Tmax - Qs)/I(1-10^{-A808})$, where h is the heat transfer coefficient, S is the surface area of the container, ΔT_{max} is the temperature change of MnEMNPs solution at the maximal steady-state temperature (40.69 ^{o}C), Qs is the heat associated with the NIR light absorbance of the pure water (measured to be 35.73 mW), I is the laser power density (2 W cm^{-2}), and A_{808} is the absorbance of MnEMNPs at 808 nm (0.82). Substituting all values into Equation 3, the η value of MnEMNPs was calculated to be 23.5%. The PTCE of MnEMNPs was remarkably higher than 13.1% for polydopamine-coated magnetic composite particle (Fe_3O_4@PDA-5),[3] 19.5% for CuS nanocrystals, and 22% for Au nanorods.[4]

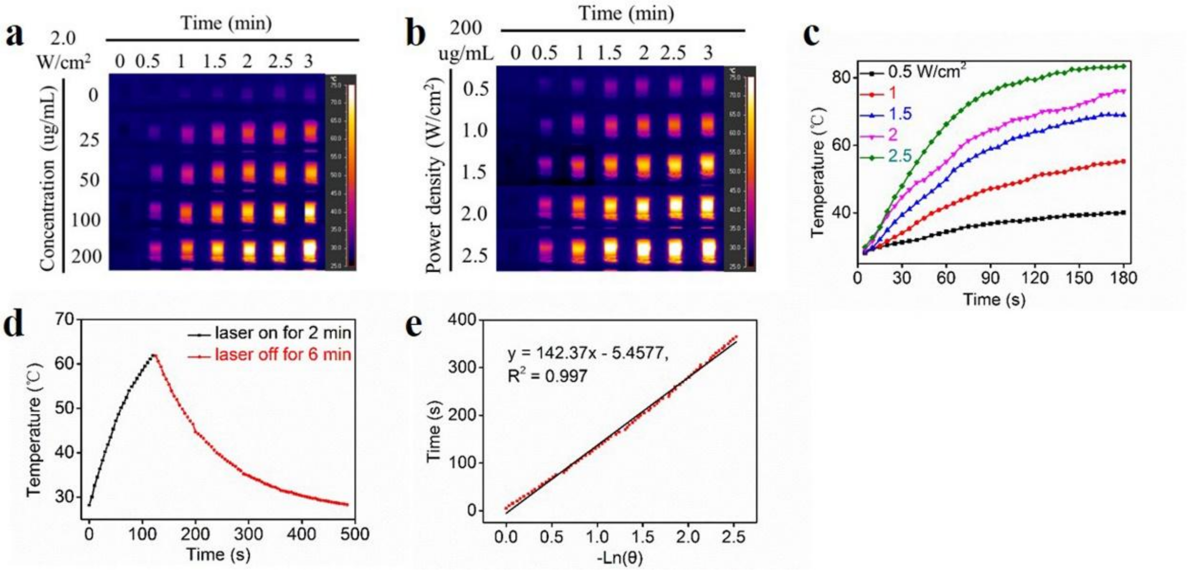

Supplementary Figure 14. Photothermal performance of MnEMNPs. (a) Infrared thermal images of MnEMNPs aqueous solutions with various concentrations as a function of irradiation time under exposure to 808 nm laser at 2 W cm^{-2}. Infrared thermal images (b) and temperature elevation (c) of 200 μg mL^{-1} MnEMNPs aqueous

solution with different power densities as a function of irradiation time with exposure to a 808 nm laser. (d) Photothermal heating curves of 50 μg mL^{-1} MnEMNPs irradiated by 808 nm laser at 2 W cm^{-2} over one laser on/off cycle (laser on for 2 min and off for 6 min). (e) Linear time data *versus* -Lnθ obtained from the cooling period of Figure d.

After longstanding laser irradiation, neither a color change nor an absorbance decrease was observed for MnEMNPs. The TEM image showed no obvious morphology or size changes. In contrast, the Au nanorods suffered a remarkable absorbance loss resulting from their melting and aggregation.

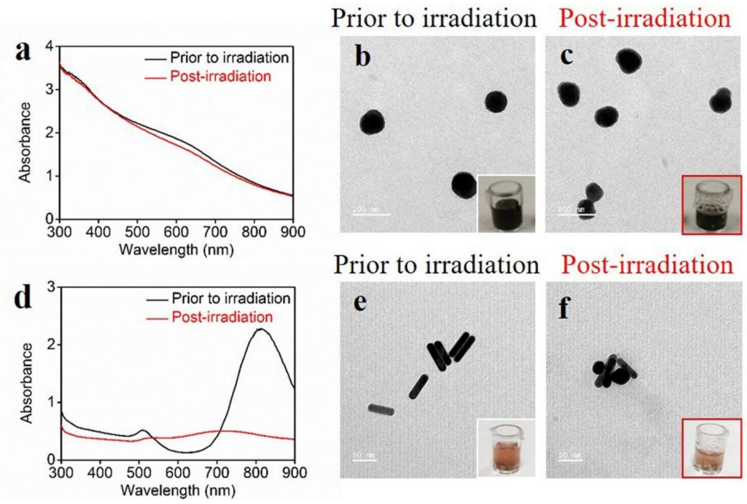

Supplementary Figure 15. Thermal stability of MnEMNPs compared with Au nanorods. UV-vis absorption spectra and TEM images of MnEMNPs (a, b, c) and Au nanorods (d, e, f) dispersed in water before and after laser irradiation (808 nm, 2 W cm^{-2}, 30 min), respectively. The insets show corresponding digital photographs of MnEMNPs and Au nanorods aqueous dispersions before and after laser irradiation.

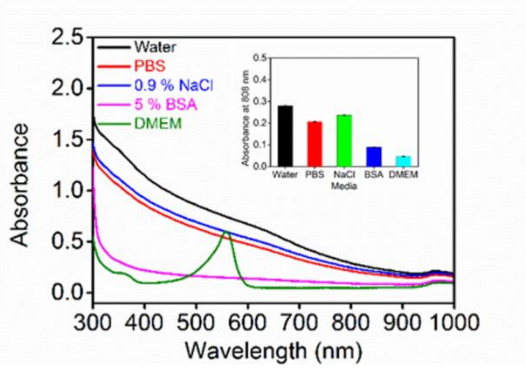

Supplementary Figure 16. UV-vis absorption spectra of MnEMNPs dispersed in various media for 24 h. The inset shows corresponding absorbance at 808 nm, respectively.

The as-prepared MnEMNPs could be dispersed in water without noticeable aggregation for more than six months indicating prominent water dispersion. However, they partially precipitated from other biological media including PBS, 0.9% NaCl, 5% BSA, and DMEM after 24 h incubation (Figure S17). In comparison, the PMnEMNPs exhibited consistent dispersion stability in these media without observable agglomeration.

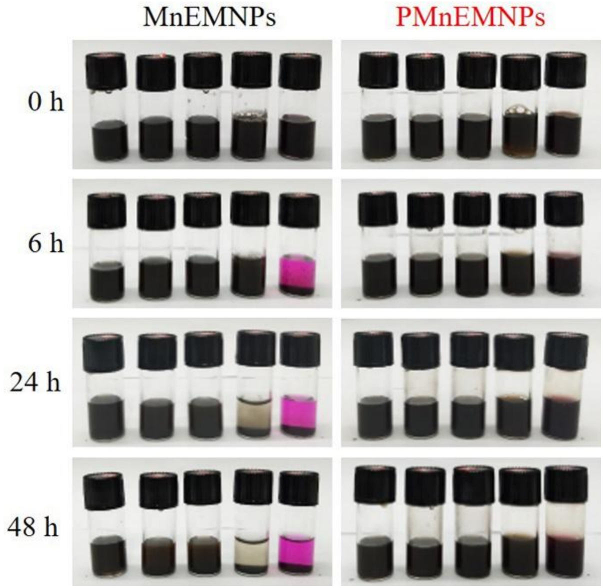

Supplementary Figure 17. Colloidal dispersion stability. Digital photographs of MnEMNPs and PMnEMNPs dispersed in various media for different times. The samples left to right were water, PBS, 0.9% NaCl, 5% BSA, and DMEM solution, respectively.

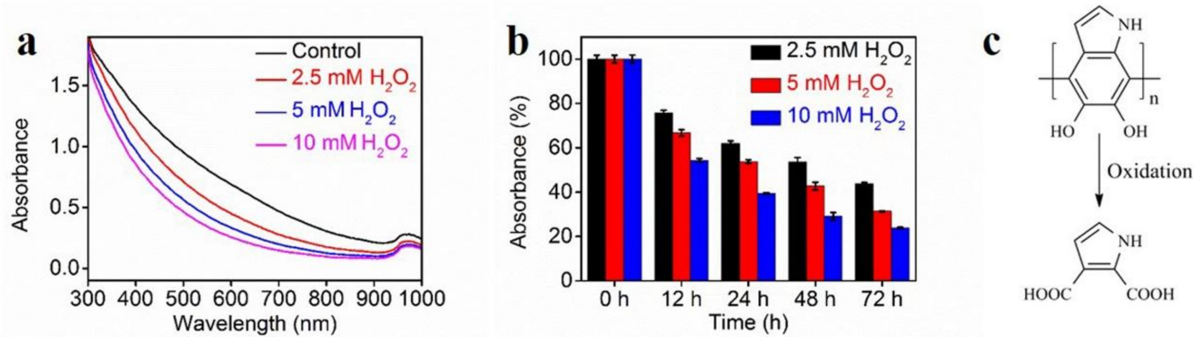

Supplementary Figure 18. H_2O_2-triggered degradation assay. (a) UV-vis absorption spectra of PMnEMNPs dispersion before and after incubation with various concentrations of H_2O_2 for 24 h. (b) Normalized absorbance change of PMnEMNPs dispersion in the presence of various concentrations of H_2O_2 at 808 nm as a function

of incubation time. The error bars are the mean values ± s.d.; n = 3. (c) Chemical degradation pathway of MnEMNPs by H_2O_2.

Serum biochemical analysis was performed for histocompatibility assessment (Figure S19). Neither hepatic nor renal function indexes exhibited significant difference at different time points after PMnEMNPs administration compared with control. This indicates that PMnEMNPs have no appreciable toxic effects on liver or kidney function.

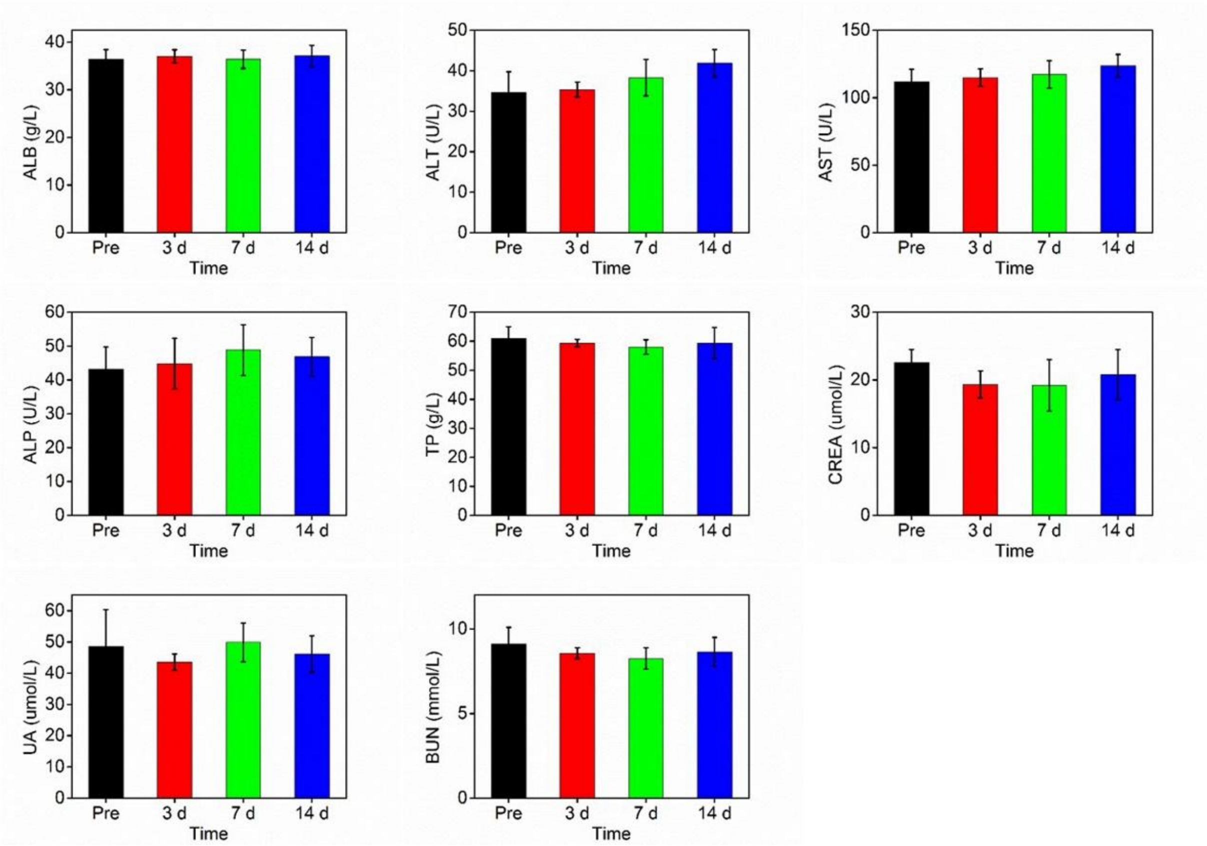

Supplementary Figure 19. Serum biochemistry indexes of mice after intravenous injection of PMnEMNPs for different days (n = 5). The important liver and kidney function indicators including albumin (ALB), alanine aminotransferase (ALT),

aspartate aminotransferase (AST), alkaline phosphatase (ALP), total protein (TP), creatinine (CREA), uric acid (UA), and blood urine nitrogen (BUN) were determined in mice that received injection of PMnEMNPs or saline control. Error bars are the mean values ± s.d.; n = 5.

H&E staining was performed to evaluate potential tissue damage induced by PMnEMNPs. As expected, there were no noticeable pathological abnormalities (such as cell necrosis and inflammatory infiltration) in the organs of mice that received different treatments compared with control (Figure S20). Furthermore, neither side effects nor behavioral abnormalities were observed.

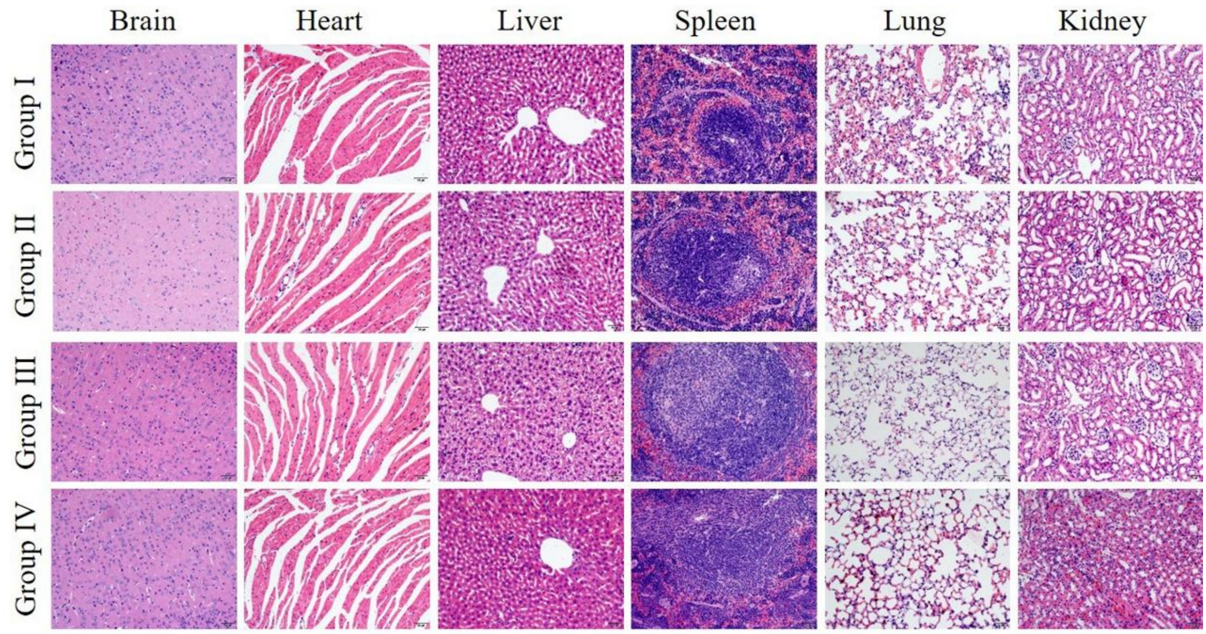

Supplementary Figure 20. H&E staining of histological sections of major organs obtained from mice at day 15 post treatment. Images were taken under 20x objective. Scale bar, 50 μm.

H_2O_2 is a common endogenous molecule in tissue-resident phagocytes.[5] We analyzed the liver because of the possible accumulation of PMnEMNPs in the mononuclear phagocyte system. PMnEMNPs provided positive and negative contrast enhancement on T_1WI and T_2WI in the liver (Figure S21), respectively. Versus baseline images, the T_1WI and T_2WI showed maximum enhanced signal at 10 min post-injection, respectively, corroborating the high accumulation of PMnEMNPs in liver. The signal then gradually recovered and was close to baseline 24 h post-injection. This might benefit from the H_2O_2-responsive decomposition process of PMnEMNPs.

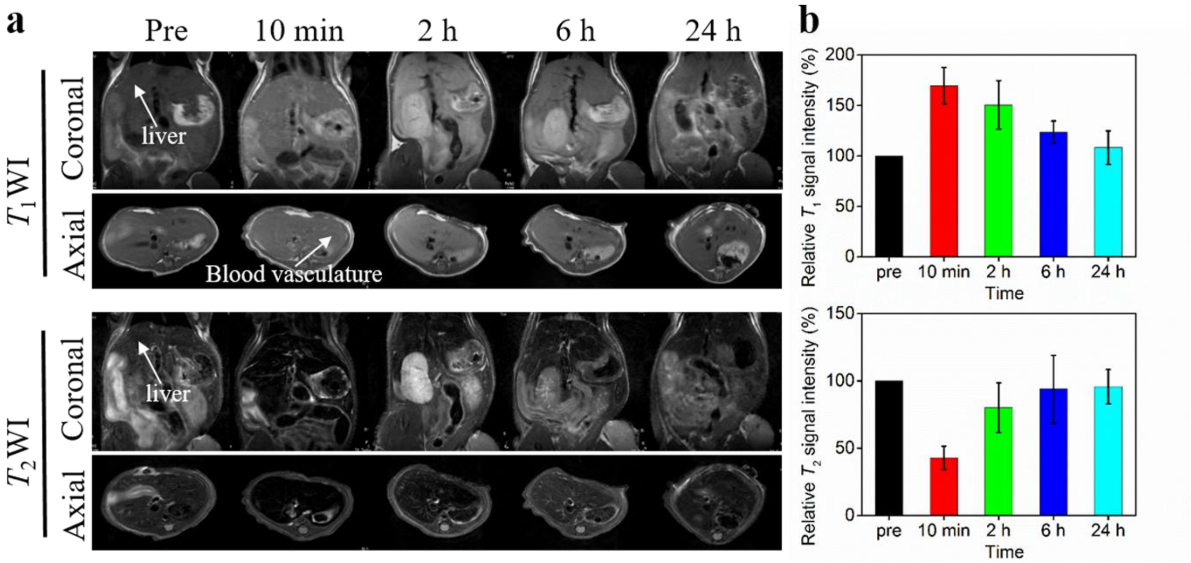

Supplementary Figure 21. PMnEMNPs serve as T_1-T_2 DMCAs *in vivo*. (a) T_1WI and T_2WI (7.0 T) of mice liver. The images were acquired before and at different time points after intravenous injection of PMnEMNPs at a dose of 73 μmol Mn kg^{-1} BW. Each acquisition contained coronal and axial planes focused on the region of interest

liver. (b) Corresponding normalized signal intensity determined from liver. Errors are the mean values ± s.d.; n = 4.

Kidneys were selected for analysis to evaluate the metabolic process of fragments disintegrated from PMnEMNPs. The kidneys exhibited gradually increased contrast enhancement with time both on T_1WI and T_2WI (Figure S22) indicating that at least a portion of the fragments could be excreted *via* the kidneys.

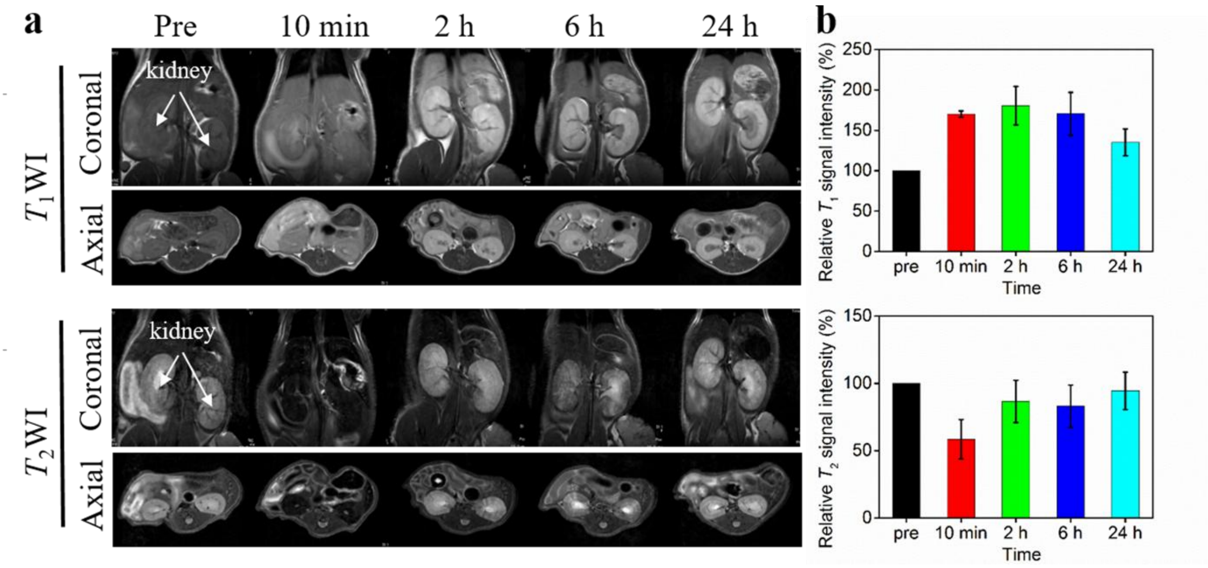

Supplementary Figure 22. PMnEMNPs serve as T_1-T_2 DMCAs *in vivo*. (a) T_1WI and T_2WI (7.0 T) of mice kidneys. The images were acquired before and at different time points after intravenous injection of PMnEMNPs at a dose of 73 μmol Mn kg^{-1} BW. Each acquisition contained coronal and axial planes focused on the kidneys' region of interest. (b) Corresponding normalized signal intensity determined from kidneys. Errors are the mean values ± s.d.; n = 4.

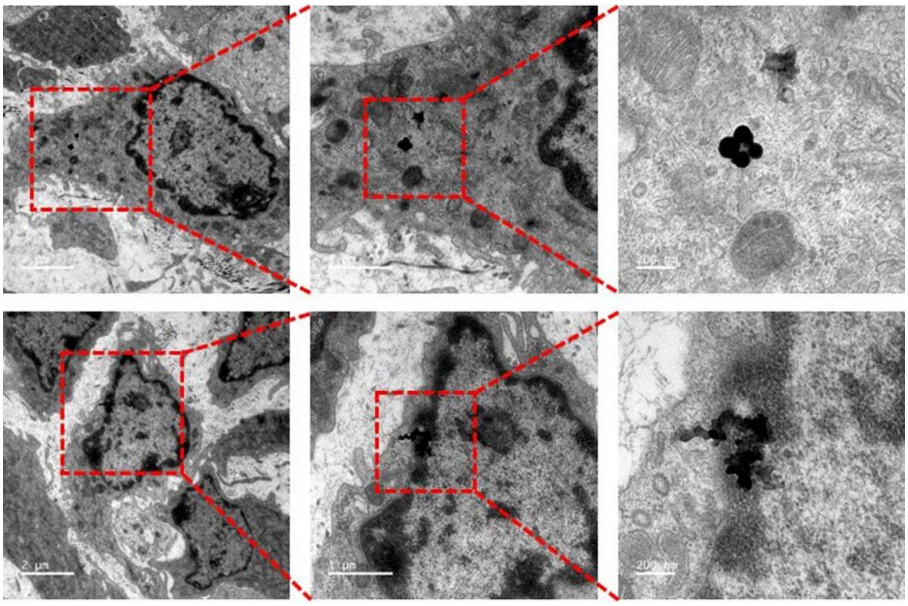

Supplementary Figure 23. Representative TEM images of tumor tissues at 2 h (upper row) and 12 h (lower row) post injection of PMnEMNPs. Scale bars: 2 μm, 1 μm and 200 nm (from left to right).

The real-time infrared thermal images demonstrated that the temperature elevation at tumor site in mice treated with PMnEMNPs positively correlated with irradiation time, while the photothermal effect in mice treated with PBS was not apparent. Upon laser irradiation, the temperature of the tumor region in the former group increased to 50.4 °C, much higher than the 39.3 °C in the latter group. This is benefitted from the strong absorption of PMnEMNPs in the NIR region and their high PTCE.

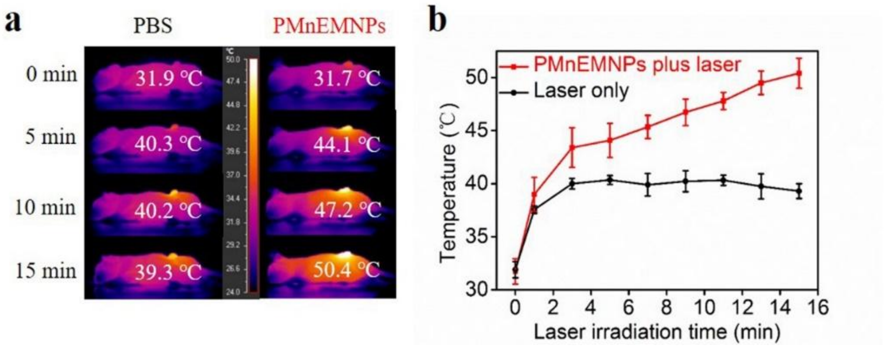

Supplementary Figure 24. *In vivo* PTT. (a) Infrared thermal images of U87MG tumor-bearing mouse post intravenous injection of PMnEMNPs or PBS, followed by 808 nm laser irradiation (2 W cm^{-2}). (b) The corresponding temperature change at tumor site during laser irradiation process. Errors are the mean values ± s.d.; n = 4.

Ki67 and TUNEL staining results demonstrated more malignant cell proliferation and less cell apoptosis in mice treated with PBS than those treated with PMnEMNPs, respectively.

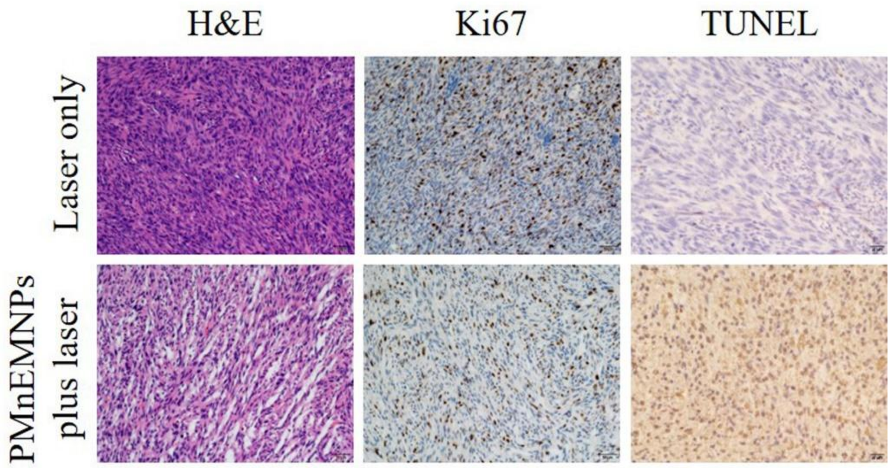

Supplementary Figure 25. Histological analysis. Representative H&E, Ki-67, and TUNEL stained histological images of tumor sections from mice that received

intravenous injection of PMnEMNPs and laser irradiation at 24 h post-injection (brown stain for Ki-67 and TUNEL, and blue for nuclei). Scale bar, 50 μm.

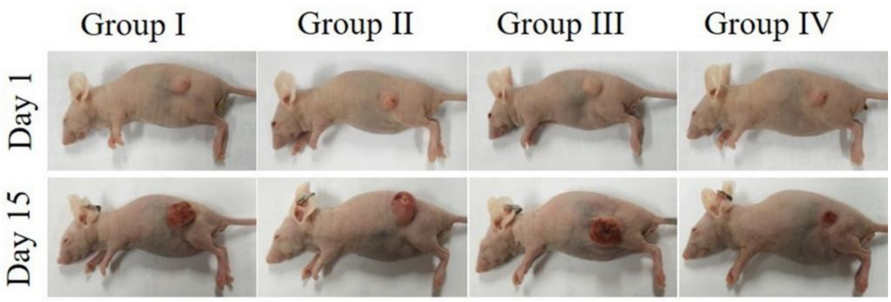

Supplementary Figure 26. Representative photographs of U87MG tumor-bearing mice before (upper row)and at day 15 (lower row) after different treatments.

Digital photographs and quantitative average weight of tumors *ex vivo* were obtained at day 15 after treatment (Figure S27), demonstrating remarkable tumor suppression effect without any regrowth following PMnEMNPs plus laser irradiation treatment.

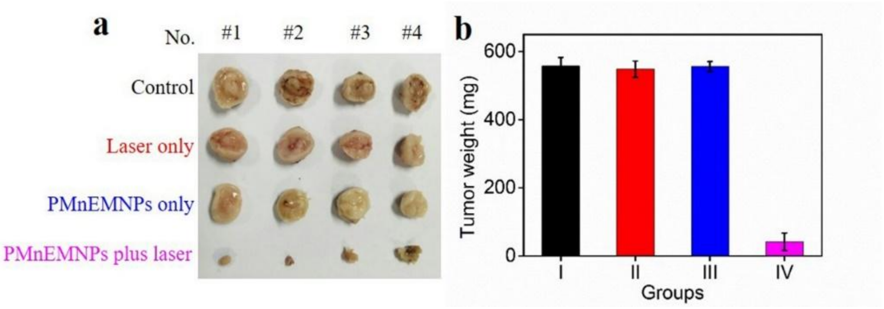

Supplementary Figure 27. *In vivo* therapeutic effect. (a) Digital photographs and (b) average weight of tumors *ex vivo* obtained at day 15 after treatment. Errors are the mean values ± s.d.; n = 4.

The yields and Mn loading efficiency of products gradually increased as the DL-DOPA/$KMnO_4$ feeding ratio ranged from 1:0.15 to 1:0.6.

Supplementary Table 1. Yields and Mn loading of products with different reaction conditions.

Reaction condition	I	II	III
DL-DOPA added (mg)	118.2	118.2	118.2
$KMnO_4$ added (mg)	14.22	28.44	56.88
DL-DOPA/$KMnO_4$ molar ratio	1:0.15	1:0.3	1:0.6
Yields (mg)	25.9±0.7	50.0±3.0	67.6±2.7
Mn loading (wt/wt, %)	9.8±0.3	10.2±0.6	13.6±0.5

Supplementary Table 2. Relaxivities of previously reported metal ions incorporated MelNPs.

**Year[ref]**	**Contrast agent**	**Metal loading (wt/wt)**	**Magnetic field**	**r_1 ($mM^{-1}s^{-1}$)**	**r_2 ($mM^{-1}s^{-1}$)**

2013[4]	Gd-DTPA-Dpa-melanin CNSs		1.5 T	6.9	
2013[1]	PEG-Fe^{3+}-MelNPs	0.72%	3.0 T	17	18
2014[6]	Fe-RGD-PEG-MNP		1.0 T	1.2	
2015[7]	M_GL_R-MNP(Fe)-HER2		0.47 T	6.7	
2015[8]	h-Au-melanin-PEG-Fe^{3+}		4.7 T	7.8	10.3
2015[9]	PMPDA NPs		9.4 T	6.55	
2016[10]	TRITC-Gd-Mel@SiO_2 NPs	5.4%	7.0 T	14.3	
2016[11]	PDA-Fe^{3+}-ICG NPs	0.83%	3.0 T	14	83.3
2016[12]	PDA-ICG-PEG-DOX(Mn)		3.0 T	14.15	39.2
2016[13]	^{64}Cu-MMNs		7.0 T		167.28
2016[14	MNP-Gd^{3+}		3.0 T	1.968	

]				4	
2016[15]	PDAs@CP_3-DOX	6.76%	1.5 T	7.524	45.92
2017[16]	MNP-PEG-Mn		3.0 T	20.56	
2017[17]	iMNP		0.47 T	6.2	
2017[18]	CDPGM		7.0 T	14.06	

Supplementary Table 3. Relaxivities of previously reported manganese-based MRI CAs.

**Year**	**Contrast agent**	**Magnetic field**	***r*₁ (mM⁻¹s⁻¹)**	***r*₂ (mM⁻¹s⁻¹)**
2002[19]	Mn(EDTA)(BOM)-HSA	0.47 T	3.6	
2008[20]	$Mn_3(BTC)_2(H_2O)_6$	9.4 T	4	112.8
2012[21]	Mn-L complex	1.5 T	3.6	
2014[22]	$[Mn2(nompa)_2(H_2O)_2](ClO_4)_2$	0.5 T	3.32	

2015[23]	Mn(III)-PDA	7.0 T	5.4	
2015[24]	$[Mn(PyC3A)(H_2O)]^-$	1.5 T	2.1	
2015[25]	CuS@MPG NPs	7.0 T	7.1	
2015[26]	HMPB-Mn	7.0 T	7.43	
2016[27]	Mn-HMSNs	7.0 T	2.46	74.17
2017[28]	Mn-LDH		9.48	
2017[29]	UMFNPs	3.0 T	8.43	

Supplementary Table 4. Relaxivities of MnEMNPs, Mn ion standards, and Gd-DTPA.

	**Magnetic field**	$\boldsymbol{r_1}$ **($mM^{-1}s^{-1}$)**	$\boldsymbol{r_2}$ **($mM^{-1}s^{-1}$)**	$\boldsymbol{r_2/r_1}$

	1.5 T	60.8	52.2	0.86
	3.0 T	36.8	82.1	2.23
**MnEMNPs**	7.0 T	14.2	145.4	10.24
	9.4 T	11.1	119.8	10.79
**Mn^{2+} ions**	1.5 T	7.6	47.5	6.25
	7.0 T	4.9	160.3	32.71
**Gd-DTPA**	1.5 T	6.8	4.5	0.66
	7.0 T	3.8	5.5	1.45

Supplementary References

[1]. K. Y. Ju, J. W. Lee, G. H. Im, S. Lee, J. Pyo, S. B. Park, J. H. Lee and J. K. Lee, *Biomacromolecules*, **2013**, *14*, 3491.

[2]. D. K. Roper, W. Ahn and M. Hoepfner, *J Phys Chem C Nanomater Interfaces*, **2007**, *111*, 3636.

[3]. R. Zheng, S. Wang, Y. Tian, X. Jiang, D. Fu, S. Shen and W. Yang, *ACS Appl Mater Interfaces*, **2015**, *7*, 15876.

[4]. Y. Liu, K. Ai, J. Liu, M. Deng, Y. He and L. Lu, *Adv Mater*, **2013**, *25*, 1353.

[5]. A. C. Cave, A. C. Brewer, A. Narayanapanicker, R. Ray, D. J. Grieve, S. Walker and A. M. Shah, *Antioxid Redox Signal*, **2006**, *8*, 691.

[6]. Q. Fan, K. Cheng, X. Hu, X. Ma, R. Zhang, M. Yang, X. Lu, L. Xing, W.

Huang, S. S. Gambhir and Z. Cheng, *J Am Chem Soc*, **2014**, *136*, 15185.

[7]. Y. I. Yoon, K. Y. Ju, H. S. Cho, K. N. Yu, J. J. Lee, G. J. Ahn, S. H. Lee, M. H. Cho, H. J. Lee, J. K. Lee and T. J. Yoon, *Chem Commun (Camb)*, **2015**, *51*, 9455.

[8]. K. Y. Ju, S. Lee, J. Pyo, J. Choo and J. K. Lee, *Small*, **2015**, *11*, 84.

[9]. Z. H. Miao, H. Wang, H. Yang, Z. L. Li, L. Zhen and C. Y. Xu, *ACS Appl Mater Interfaces*, **2015**, *7*, 16946.

[10]. S. Cho, W. Park and D. H. Kim, *ACS Appl Mater Interfaces*, **2017**, *9*, 101.

[11]. D. Hu, C. Liu, L. Song, H. Cui, G. Gao, P. Liu, Z. Sheng and L. Cai, *Nanoscale*, **2016**, *8*, 17150.

[12]. Z. Dong, H. Gong, M. Gao, W. Zhu, X. Sun, L. Feng, T. Fu, Y. Li and Z. Liu, *Theranostics*, **2016**, *6*, 1031.

[13]. J. Lin, M. Wang, H. Hu, X. Yang, B. Wen, Z. Wang, O. Jacobson, J. Song, G. Zhang, G. Niu, P. Huang and X. Chen, *Adv Mater*, **2016**, *28*, 3273.

[14]. W. W. Cai, L. J. Wang, S. J. Li, X. P. Zhang, T. T. Li, Y. H. Wang, X. Yang, J. Xie, J. D. Li, S. J. Liu, W. Xu, S. He, Z. Cheng, Q. L. Fan and R. P. Zhang, *J Biomed Mater Res A*, **2017**, *105*, 131.

[15]. Y. Chen, K. Ai, J. Liu, X. Ren, C. Jiang and L. Lu, *Biomaterials*, **2016**, *77*, 198.

[16]. W. Xu, J. Sun, L. Li, X. Peng, R. Zhang and B. Wang, *Biomater Sci*, **2017**, DOI: 10.1039/c7bm00635g.

[17]. S. W. Ha, H. S. Cho, Y. I. Yoon, M. S. Jang, K. S. Hong, E. Hui, J. H. Lee and

T. J. Yoon, *J Nanobiotechnology*, **2017**, *15*, 73.

[18]. S. Wang, J. Lin, Z. Wang, Z. Zhou, R. Bai, N. Lu, Y. Liu, X. Fu, O. Jacobson, W. Fan, J. Qu, S. Chen, T. Wang, P. Huang and X. Chen, *Adv Mater*, **2017**, *29*.

[19]. S. Aime, L. Anelli, M. Botta, M. Brocchetta, S. Canton, F. Fedeli, E. Gianolio and E. Terreno, *J Biol Inorg Chem*, **2002**, *7*, 58.

[20]. K. M. Taylor, W. J. Rieter and W. Lin, *J Am Chem Soc*, **2008**, *130*, 14358.

[21]. H. Su, C. Wu, J. Zhu, T. Miao, D. Wang, C. Xia, X. Zhao, Q. Gong, B. Song and H. Ai, *Dalton Trans*, **2012**, *41*, 14480.

[22]. E. Molnar, N. Camus, V. Patinec, G. A. Rolla, M. Botta, G. Tircso, F. K. Kalman, T. Fodor, R. Tripier and C. Platas-Iglesias, *Inorg Chem*, **2014**, *53*, 5136.

[23]. A. Barandov, B. B. Bartelle, B. A. Gonzalez, W. L. White, S. J. Lippard and A. Jasanoff, *J Am Chem Soc*, **2016**, *138*, 5483.

[24]. E. M. Gale, I. P. Atanasova, F. Blasi, I. Ay and P. Caravan, *J Am Chem Soc*, **2015**, *137*, 15548.

[25]. R. Liu, L. Jing, D. Peng, Y. Li, J. Tian and Z. Dai, *Theranostics*, **2015**, *5*, 1144.

[26]. X. Cai, W. Gao, M. Ma, M. Wu, L. Zhang, Y. Zheng, H. Chen and J. Shi, *Adv Mater*, **2015**, *27*, 6382.

[27]. L. Yu, Y. Chen, M. Wu, X. Cai, H. Yao, L. Zhang, H. Chen and J. Shi, *J Am Chem Soc*, **2016**, *138*, 9881.

[28]. B. Li, Z. Gu, N. Kurniawan, W. Chen and Z. P. Xu, *Adv Mater*, **2017**, DOI: 10.1002/adma.201700373.

[29]. H. Zhang, L. Li, X. L. Liu, J. Jiao, C. T. Ng, J. B. Yi, Y. E. Luo, B. H. Bay, L. Y. Zhao, M. L. Peng, N. Gu and H. M. Fan, *ACS Nano*, **2017**, *11*, 3614.
